# Function and Role of Histamine H_1_ Receptor in the Mammalian Heart

**DOI:** 10.3390/ph16050734

**Published:** 2023-05-11

**Authors:** Joachim Neumann, Britt Hofmann, Uwe Kirchhefer, Stefan Dhein, Ulrich Gergs

**Affiliations:** 1 Institut für Pharmakologie und Toxikologie, Medizinische Fakultät, Magdeburger Straße 4, Martin-Luther-Universität Halle-Wittenberg, 06097 Halle, Germany; ulrich.gergs@medizin.uni-halle.de; 2 Herzchirurgie, Medizinische Fakultät, Martin-Luther-Universität Halle-Wittenberg, Ernst-Grube Straße 40, 06097 Halle, Germany; britt.hofmann@uk-halle.de; 3 Institut für Pharmakologie und Toxikologie, Domagkstraße 12, Westfälische Wilhelms-Universität, 48149 Münster, Germany; kirchhef@uni-muenster.de; 4 Rudolf-Boehm Institut für Pharmakologie und Toxikologie, Härtelstraße 16–18, Universität Leipzig, 04107 Leipzig, Germany; stefan.dhein@medizin.uni-leipzig.de

**Keywords:** histamine, histamine H_1_ receptor, human heart, signal transduction

## Abstract

Histamine can change the force of cardiac contraction and alter the beating rate in mammals, including humans. However, striking species and regional differences have been observed. Depending on the species and the cardiac region (atrium versus ventricle) studied, the contractile, chronotropic, dromotropic, and bathmotropic effects of histamine vary. Histamine is present and is produced in the mammalian heart. Thus, histamine may exert autocrine or paracrine effects in the mammalian heart. Histamine uses at least four heptahelical receptors: H_1_, H_2_, H_3_ and H_4_. Depending on the species and region studied, cardiomyocytes express only histamine H_1_ or only histamine H_2_ receptors or both. These receptors are not necessarily functional concerning contractility. We have considerable knowledge of the cardiac expression and function of histamine H_2_ receptors. In contrast, we have a poor understanding of the cardiac role of the histamine H_1_ receptor. Therefore, we address the structure, signal transduction, and expressional regulation of the histamine H_1_ receptor with an eye on its cardiac role. We point out signal transduction and the role of the histamine H_1_ receptor in various animal species. This review aims to identify gaps in our knowledge of cardiac histamine H_1_ receptors. We highlight where the published research shows disagreements and requires a new approach. Moreover, we show that diseases alter the expression and functional effects of histamine H_1_ receptors in the heart. We found that antidepressive drugs and neuroleptic drugs might act as antagonists of cardiac histamine H_1_ receptors, and believe that histamine H_1_ receptors in the heart might be attractive targets for drug therapy. The authors believe that a better understanding of the role of histamine H_1_ receptors in the human heart might be clinically relevant for improving drug therapy.

## 1. Introduction

Histamine (=2-(1H-imidazole-4-yl)-ethanamine [1]) is an essential endogenous amine in mammals. Consistent with this, at least one has measured considerable histamine concentrations in the lung, skin, heart and intestinal tract of mammals and humans [1,2,3].

For instance, in the human ventricle and human kidney, approximately 45–55 µM histamine has been reported [1,2,3]. Histamine probably affects all cells and all organs of mammals, including humans. The roles and actions of histamine include the following:

- Histamine acts as a neurotransmitter in the central nervous system, but also plays a role as a neurotransmitter in the gut.
- Histamine has many effects on the skin and cutaneous immune system.
- Histamine induces the weal and flare reaction in the skin [1].
- Histamine is involved in contractions of the intestinal, but also vascular smooth muscle, leading to dilatation of capillaries and enhancing acid secretion in the stomach.
- Histamine induces bronchoconstriction, especially in allergic asthma, but also in anaphylactic reactions in the cardiovascular system.
- Other widely known effects of histamine include itching, rhinorrhoea (by stimulating secretory glands), and sneezing.
- Most relevant in this context, histamine contributes to the regulation of cardiac contraction [1].

As shown in Table 1, histamine induces these various cellular effects via at least four guanosine-triphosphate-binding-protein (G protein)-coupled receptors (histamine H_1_, H_2_, H_3_, and H_4_ receptors) [1]. This study was solely concerned with the histamine H_1_ receptor in the heart (histamine H_1_ receptors in other organs [1,4]). Histamine H_1_ receptors occur in the entire body of most mammals, for example, in the central nervous system, immunological cells, muscle cells in the gut, and the heart [1]. Specifically in the heart, histamine H_1_ receptors are found in several cell types, such as cardiomyocytes, endothelial cells, fibroblasts, smooth muscle cells, nerve cells, and mast cells [1,3,5]. The activation of histamine H_1_ receptors takes part in many processes [1].

Commonly accepted symptoms of activation of histamine H_1_ receptors, in general, are itching in the skin, sneezing, and other symptoms of hay fever [1,3]. Hay fever is the most common indication for using histamine H_1_ receptor antagonistic drugs [1]. Worldwide, histamine H_1_ receptor antagonists are among the most frequently used drugs that underscore the need to better understand the histamine H_1_ receptor in the human heart [4,6]. Histamine H_1_ receptor antagonists are currently used for additional indications in dermatology, such as in some cases of urticaria [6]. Older antagonists at the histamine H_1_ receptor, such as diphenhydramine, are sold over the counter as sedatives without prescriptions. These older drugs have sedative side effects explained by their ability to pass the blood–brain barrier to block histamine H_1_ receptors in the central nervous system [7]. In this case, an adverse side effect (sedation) was repurposed as an indication. This can be viewed with some scepticism. As concerns our present topic, there is, for instance, evidence that histamine H_1_ receptor antagonists, in vivo, can slow depolarisation in the sinus node or atrioventricular node. The inhibitory effects of histamine H_1_ receptor stimulation in the sinus node and atrioventricular node are antagonised by histamine H_1_ receptor antagonistic drugs.

Thus, histamine in the heart can stimulate histamine H_2_ receptors unopposed, leading directly to a faster heart beat and more rapid atrioventricular node signal transduction [8]. In addition, some older antidepressant drugs (amitriptyline) and some antipsychotic drugs (haloperidol) are also potent histamine H_1_ receptor antagonists, at least on receptors in transfected cells [9]. Whether they antagonise the effects of histamine on histamine H_1_ receptors in the heart remains to be studied in more depth. It is known that promethazine antagonises the positive inotropic effect of histamine via histamine H_1_ receptors in isolated guinea pig left atrial preparations [10,11]. Promethazine is an old drug used as an antipsychotic agent but is no longer clinically relevant. Furthermore, histamine H_1_ receptors and histamine as agonists in the human heart should play a physiological role. Histamine H_1_ receptor density, function, and the concentration of histamine can be altered in cardiac disease.

**Table 1:** Typical histamine H_1_ receptor agonists are useful in cardiovascular research. The IC_50_ values are presented as they were published in the references. pKi is the negative decadic logarithm of the molar concentration with 50% inhibition of the ligand binding or other functional effects. Other publications reported the nano mole per liter (nM) concentration with 50% inhibition of ligand binding or other functional effects. For the planning of studies, what potent off-target effects might interfere (binding to other receptors or structures) is also relevant. Superscript numbers in the columns refer to references.

	H_1_R Affinity pKi	H_2_R Affinity pKi	Off-Target Effect	References
2-Methylhistamine	17	4		Hill et al., 1997 [12]
2-(2-Pyridyl)ethylamine (PEA)	5.6	2.5		Hill et al., 1997 [12]
2-(2-Thiazolyl) ethyl amine (ThEA)	^1^ 5.3, ^2^ 26	^2^ 2.2	^3^ Release of noradrenaline in the human heart	^1^ Panula et al., 2015 [1] ^2^ Hill et al., 1990, 1997 [10,12] ^3^ Own unpublished data
8-S-Lisuride	pKi 7.27		Dopamine-, serotonin-, adrenergic receptors,	Pertz et al., 2006 [11]
Bromocriptine	pKi 5.72		Dopamine receptors	Pertz et al., 2006 [11]
Histamine	^1^ 4.6	^1^ 5.6	^2^ Release of noradrenaline from mouse heart	^1^ Panula et al., 2015 [1] ^2^ Gergs et al., 2019 [13]
Histaprodifen	5.7			Panula et al., 2015 [1]
Methylhistaprodifen	27.1 nM			Carman-Krzan et al., 2003 [14]
Suprahistaprodifen	^1^ 4.3 nM		^2^ Release of noradrenaline in the mouse und human heart	^1^ Carman-Krzan et al., 2003 [14] ^2^ Own unpublished data
trans-PAT	1.15 nM			Moniri et al., 2004 [15]

Note: In Hill et al., 1997 [12], agonist ratios are given. Superscript numbers refer to numbers of the references in the same row.

## 2. Histamine H_1_ Receptor Structure

Two groups cloned the human histamine H_1_ receptor [16,17]. It contains 487 amino acids. In early Northern blots of human tissue, mRNA for the histamine H_1_ receptor was detected in the human heart [17]. The human gene for the histamine H_1_ receptor is localised to chromosome 3p14-p21 [18]. A phylogenetic tree indicates substantial differences between human and mouse sequences for the histamine H_1_ receptor [19]. The homologies of the rat, mouse and guinea pig histamine H_1_ receptor to the human histamine H_1_ receptor were 87.8%, 84% and 82.9%, respectively. The sequence differences of the histamine H_1_ receptor between humans and guinea pigs (often used species in histamine research) are significant enough that there are subtle changes in pharmacology. The affinities of agonists and antagonists are, in some examples, functionally different (GTPase activity assays) between humans and guinea pigs. This underscores the need to primarily use human histamine H_1_ receptor model systems. Mice with ablation of histamine H_1_ receptors have been published [20]. However, the cardiac phenotype was not changed compared to wild-type mice concerning, e.g., heart rate [21]. The crystal structure of the histamine H_1_ receptor as a complex with the histamine H_1_ receptor-antagonist doxepin (Table 2) was reported [22]. In this structure, three sites were described where doxepin was in contact with the histamine H_1_ receptor, namely transmembrane domains 3, 5 and 6.

Some promoter studies for the human histamine H_1_ receptor have been published in HeLa cells (an epithelial carcinoma cell line). The following transcription factors in the promoter were functionally found: Ets-1, AP-1, PARP-1, Ku86, and Ku70 [23]. By inspection of the human promoter region of the histamine H_1_ receptor, others described binding sites in the DNA for mini-Zinc finger 1, heat shock transcription factor-2, signal transducer, and activator of transcription 5A and the glucocorticoid receptor [24]. These promoter motifs are expected to be used in the human heart to alter the expression of the histamine H_1_ receptor under drug therapy (e.g., glucocorticoids) or diseased states (e.g., sepsis). A more detailed analysis of the role of the promoter of the histamine H_1_ receptor is mandated. In the human histamine H_1_ receptor gene, at least 88 functionally relevant single nucleotide polymorphisms have been detected [25]. Whether these polymorphisms are relevant in the human heart is presently unknown, and an obvious research need. However, a point mutation F435A reduced the affinity of the histamine H_1_ receptor for histamine [26]. If this or a functionally similar mutation occurs in the human heart, a reduced mechanical and electrophysiological role of the histamine H_1_ receptor in the human heart will follow.

**Table 2:** Typical histamine H_1_ receptor antagonists are useful in cardiovascular research and their (less potent) effects on histamine H_2_ receptors. The IC_50_ values are presented as they were published in the references. pK_i_ is the negative decadic logarithm of the molar concentration with 50% inhibition of the ligand binding or other functional effects. Other publications reported the nano mole per liter (nM) concentrations with 50% inhibition of the ligand binding or other functional effects. For the planning of studies, what potent off-target effects might interfere (binding to other receptors) is also relevant. pK_B_: negative decadic logarithm of the inhibition constant from the GTPase assay [9]. R- and S-refer to the enantiomers of dimethindene. H_1,2_R affinity: antagonist affinity at the histamine H_1,2_ receptor. pA_2_: affinity based on functional studies using Schild plots.

	H_1_-R Affinity IC_50_ Values If Not Stated Otherwise	H_2_-R Affinity IC_50_ Values, If Not Stated Otherwise	Off-Target Effect	References
Amitryptiline	pKi 9.04	pK_B_ 6.95	antiserotoninergic	Appl et al., 2011 [9]
Astemizol	8 nM	Not done	anticholinergic	Kubo et al., 1987 [27]
(+) Chlorpheniramine	0.4 nM	1.2 µM		Hill et al., 1997 [12]
Cetirizine	pKi 7.5	Not done		Hill et al., 1997 [12]
Chlorpromazine	1.2 nM	5.9 µM	anticholinergic	Hill et al., 1997 [12]
Clemastine	0.26 nM	Not done	anticholinergic	Kubo et al., 1987 [27]
Clozapine	8.65	6.82	anticholinergic, antiadrenergic, antiserotinergic	Panula et al., 2015 [1]
Diphenhydramine	7.89	5.80	anticholinergic	Panula et al., 2015 [1]
Dimet(h)indene	R: pA_2_: 9.54 S: pA_2_: 7.86	Not done	anticholinergic, antiadrenergic, antiserotinergic	Nicholson et al., 1991 [28], Pfaff et al., 1995 [29]
Doxepin	0.06 nM	Not done	anticholinergic, antiadrenergic, antiserotinergic	Hill et al., 1997 [12]
Fluphenazine	pKi 8.25	pK_B_ 4.78	antiserotoninergic dopamine receptor	Appl et al., 2011 [9]
Haloperidol	pKi 5.71	pK_B_ 5.94	dopamine receptor	Appl et al., 2011 [9]
Imipramine	pKi 8.12	pKi 6.26	anticholinergic	Appl et al., 2011 [9]
Ketotifen	1.02 nM	Not done	anticholinergic, antiserotinergic	Feng et al., 2020 [30]
Mepyramine = pyrilamine	8.80	4.63	anticholinergic	Panula et al., 2015 [1]
Mianserin	pKi 8.92	pKi 6.36	antiadrenergic, antiserotinergic	Appl et al., 2011 [9]
Olanzapine	pKi 8.52	pK_B_ 6.02	anticholinergic, antidopaminergic, antiserotinergic	Appl et al., 2011 [9]
Perphenazine	pKi 8.59	pK_B_ 5.55	anticholinergic, antidopaminergic, antiserotinergic	Appl et al., 2011 [9]
Promethazine	1.2 nM	3.0 µM	anticholinergic	Hill et al., 1997 [12]
Terfenadine	^1^ 7.92	Not done	^2^ anticholinergic	^1^ Panula et al., 2015 [1] ^2^ Gillard et al., 2003 [31]
Triprolidine	0.2 µM	Not done	anticholinergic, antiadrenergic, antiserotinergic	Carman-Krzan 1986 [32], Nicholson et al., 1991 [28], Pfaff et al., 1995 [29]

Superscript numbers refer to numbers of the references in the same row.

## 3. Histamine H_1_ Receptor and Signal Transduction

**Figure 1:**
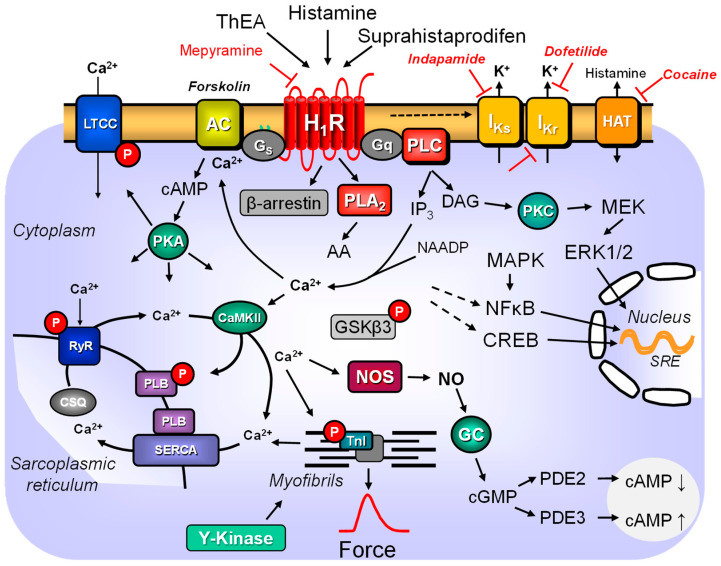
Proven signal transduction pathways for the histamine H_1_ receptor in principle. Histamine H_1_ receptors convey their effects primarily via GTP binding proteins. This leads to stimulation of PLC activity. Histamine H_1_ receptors can activate also phospholipase A2 (PLA2), leading to the formation of arachidonic acid (AA). After that, inositoltrisphosphate (IP_3_) and diacylglycerol (DAG) are formed. DAG activates protein kinase C (PKC). PKC then phosphorylates target proteins and starts a cascade with other kinases called MEK and ERK. IP_3_ acts on IP_3_ receptors. [34]. Ca^2+^ activates Ca^2+^-dependent proteins; e.g., Ca^2+^ calmodulin-dependent protein kinase II (CamKII) and the stimulation of adenylyl cyclases. cAMP-dependent protein kinase (PKA) can phosphorylate phospholamban (PLB), the ryanodine receptor (RYR), the L-type Ca^2+^-channel (LTCC), the transcription factor CREB, and the inhibitory subunits of troponin (TnI). In the SR, Ca^2+^ binds to calsequestrin (CSQ) and exits the SR via RYR. Ca^2+^ can activate nitric oxide (NO) synthases (NOS), stimulate guanylyl cyclases (GC). cGMP can activate or inhibit phosphodiesterases (PDEs. Histamine H_1_ receptors might also activate tyrosine kinases (Y-kinase. Histamine H_1_ receptors can inhibit (*I*_Kr_) or activate (*I*_Ks_) potassium channels (K) in the sarcolemma. Histamine H_1_ receptors can induce early response genes, but also via altered gene transcription (via MAPK and NFkappaB).

One can distinguish between short-term and long-term effects concerning the signal transduction of the histamine H_1_ receptor. We shall first concentrate on the short-term effects of the activation of histamine H_1_ receptors. In immediate signal transduction, several pathways can be distinguished (Figure 1). In a cell line-dependent manner, one or more of these pathways can be used. In brief, one can discern a stimulation of phospholipase C (PLC)- or phospholipase D (PLD)-activity, an increase in phospholipase A2 (PLA2)-activity, an augmentation in 3′,5′-cyclic adenosine monophosphate (cAMP), 3′,5′-cyclic guanosine monophosphate (cGMP) and a rise in Ca^2+^-transients (Figure 1). These signal transductions are initially due to coupling the histamine H_1_ receptor to one or more G proteins that are probably interconnected. Moreover, non-canonical activation of β-arrestins, independent of G proteins, also occurs, at least in myometrial cells [33], but should be looked for also in cardiomyocytes as in other cell types present in the human heart. Over time (hours or days), subsequent changes in gene transcription occur through the histamine H_1_ receptor-dependent activation of transcription factors (Figure 1). These long-term effects (requiring more than approximately 60 min) probably stem from the initial (within minutes) signal transduction steps.

Of relevance here, the histamine H_1_ receptor is constitutively active, even without histamine [35]. The situation in the heart is certainly more subtle; it may be that the receptor is, per se, active. However, this is not easy to measure because histamine is detected in the cytosol [36]. It is likely that histamine can even be produced in cardiomyocytes. Hence, to address this issue clearly, one must study mice with knock-out of the histidine decarboxylase. The histamine-dependent increase in Ca^2+^ transients in cultured cells varied from cell line to cell line, even if they contained endogenous histamine H_1_ receptors [37]. When Chinese hamster ovary (CHO) cells were transfected with the human histamine H_1_ receptor, radioligand studies noted nearly eightfold overexpression compared to human astrocytoma 132N1 cells [37]. This increase in histamine H_1_ receptor density led to a more potent effect of histamine in raising intracellular Ca^2+^ transients. In other words, increased histamine H_1_ receptor density in cells and by extrapolation in cardiomyocytes is expected to lead to more potent signal transduction.

Histamine H_1_ receptors could lead cell lines to cell proliferation (adenocarcinoma: [38]) via PLC-mediated activation. This, however, depends on the cell type and the exact signal transduction. In contrast, there are examples that histamine H_1_ receptors can also inhibit the proliferation of, for example, U937 cells [39,40]. Histamine H_1_ receptors mainly (if they use the canonical pathway) act via guanosine triphosphate-binding proteins (G proteins). Histamine H_1_ receptors couple at least via G_i/o_ [41] or G_q/11_ proteins [42]. In the neuronal NGl08-15 cell line, this stimulation was increased by a nonhydrolyzable GTP derivate and attenuated by antibodies against the alpha_11_ or alpha_q_ subunits of G proteins [42]. Moreover, histamine H_1_ receptor stimulation activates phospholipase C, increasing the intracellular levels of 1,2-diacylglycerol (DAG) und IP_3_ (inositol-1,2,4-trisphosphate). DAG can stimulate protein kinase C, and this kinase can phosphorylate regulatory proteins, such as myofilaments, and thus increase their activity [43]. Moreover, IP_3_ can release Ca^2+^ from intracellular stores (e.g., on nuclear membranes in cardiomyocytes [34]). This Ca^2+^ can activate Ca^2+^-binding proteins, for instance, in the myofilaments or Ca^2+^-calmodulin-dependent protein kinases or Ca^2+^-calmodulin-dependent phosphatases (calcineurin, PP2B) or nitric oxide (NO)-synthase (Figure 1). NO synthase leads to the formation of NO. NO can then act in an autocrine or paracrine way.

In any case, NO might activate GC in these cardiomyocytes or surrounding smooth muscle cells, endothelial cells, or fibroblasts (et cetera), leading to enhanced cGMP levels. It is well known that histamine H_1_ receptor stimulation also elevates 3′,5′-cyclic guanosine monophosphate (cGMP) levels, for instance, in rabbit cardiac preparations [44]. This cGMP can then exert several effects: activating phosphodiesterase II or inhibiting phosphodiesterase III. This is predicted to decrease or increase cAMP levels and subsequently reduce or raise the activity of PKA and force (Figure 1). One would predict that inhibitors of NO synthases or phosphodiesterase III should alter the contractile effects of histamine H_1_ receptors. Stimulatory or inhibitory cGMP analogues might also alter the contractile effects of histamine H_1_ receptors. The effect of cardiac histamine H_1_ receptors might be further altered by inhibitors of Ca^2+^ release or inhibitors of the Ca^2+^–calmodulin complex (Figure 1 for pathways, Table 3).

There are reliable experimental data that argue against the role of IP_3_ in the positive inotropic effect of histamine H_1_ receptor stimulation in the heart. The increase in IP_3_ occurred not before but after the increase in the force of contraction [54]. Moreover, it was possible to inhibit any increase in IP_3_ via PLC inhibitors, such as 2-nitro-4-carboxyphenyl-N,N-diphenylcarbamate, or neomycin, without blunting the positive inotropic effect of histamine H_1_ receptor stimulation [81,82]. Moreover, IP_3_ concentrations were raised from similar basal values using histamine H_1_ receptor stimulation to similar values using 100 µM histamine in ventricular preparations from guinea pigs and rabbits. This finding is hard to reconcile with the knowledge that the inotropic effect in the rabbit ventricle is mainly mediated by histamine H_1_ receptors and the inotropic effect of histamine in the guinea pig ventricle is only minimally mediated by histamine H_1_ receptors but mainly by histamine H_2_ receptors [83] (Table 4).

Others presented data against a connection between histamine H_1_ receptor, cGMP, and force of contraction. One study argued that because histamine H_1_ receptor stimulation elevates cGMP in cardiac tissues in which not histamine H_1_ but H_2_ receptors mediate and increase the force of contraction (e.g., left atrium of the rabbit: Table 4), a causal link between cGMP and force might be unlikely. The cGMP elevation might have occurred in non-cardiomyocytes, not cardiomyocytes, in complex tissues such as isolated atrial strips composed of several cell types [82,104]. Moreover, in the right papillary muscle of the rabbit, cGMP seems not to be required for the histamine H_1_ receptor-induced positive inotropic effect. If one gives 10 μM methylene blue to inhibit the enzymatic activity of GC (Figure 1, Table 3), the histamine H_1_ receptor-induced positive inotropic effect of histamine remained unaltered [54]. Moreover, the extent of the increase in IP_3_ concentrations after incubation with 10 µM histamine was less than 10% as the increase in IP_3_ after 10 µM carbachol, which the authors interpreted as evidence against a relevant role of IP_3_ for the signal transduction of histamine H_1_ receptors in the rabbit heart [54]. This finding would also argue against a causal link between histamine H_1_ receptors, IP_3,_ and force of contraction [83]. We would like to see these experiments repeated with isolated primary left and right atrial and ventricular cardiomyocytes from guinea pigs and rabbit hearts to fully understand the role of these putative second messengers (Figure 1).

The positive inotropic effect of histamine in the left atrium of the guinea pig was solely histamine H_1_ receptor-mediated (Table 4). Thus, the guinea pig left atrium probably presents a valid model for the histamine H_1_ receptor. Here, the signal transduction might even be biphasic, suggesting to the authors that two independent signal transduction systems are active. This conclusion was based on the experimental finding that the second phase of the positive inotropic effect of histamine H_1_ receptor stimulation in guinea pig left atrial preparations was inhibitable by 2 mM Ni^2+^ [64]. The first phase, in contrast, was not inhibitable.

In rabbits, the histamine H_1_ receptor mediates positive inotropic and positive chronotropic effects in isolated left and right atrial preparations, respectively. In contrast, in the rabbit right ventricle (papillary muscle), the histamine H_1_ receptor mainly mediated the positive inotropic effect. At the same time, there was a small (approximately 10%) positive inotropic effect mediated by histamine H_2_ receptors [54] (Table 4). Interestingly, this positive inotropic effect of histamine in the right papillary muscle of rabbits is not reduced by carbachol. This effect of carbachol, an M_2_ muscarinic receptor stimulatory analogue of acetylcholine, tests whether an inotropic effect involves cAMP. If cAMP is involved, carbachol presumably inhibits cAMP formation by inhibiting adenylyl cyclase activity [59]. This holds true for the positive inotropic effects of β-adrenoceptor stimulation or phosphodiesterase inhibition. Thus, the authors interpreted the lack of effect of carbachols as suggesting that histamine H_1_ receptors do not increase functionally relevant cAMP compartments in the rabbit ventricle [54].

As stated above, the histamine H_1_ receptor can activate PKC (Figure 1). At least in smooth muscle cells, but not yet shown in cardiomyocytes, PKC can phosphorylate and activate a phosphatase inhibitor called CPI or CPI-17 (PKC-potentiated inhibitory protein of 17 kDa). Activated CPI can reduce the activity of phosphatase 1, specifically for myosin light chains [107,108]. Moreover, if histamine H_1_ receptors elevate cAMP levels, PKA may phosphorylate and activate the protein phosphatase 1 (PP1) inhibitor 1 or DARPP 32, which would behave similar to CPI and inhibit the enzymatic activity of PP1 [109]. Moreover, PKA can phosphorylate and activate subunits of PP2A, which might alter PP2A activity [109]. Finally, there are data that cardiac histamine H_1_ receptors in the heart can stimulate tyrosine kinases (Figure 1) and thus increase tyrosine phosphorylation in the contractile proteins. This phosphorylation increased the sensitivity of the myofilament for Ca^2+^ and, thereby, forced contraction [80]. However, the exact biochemical pathways were never studied or defined. At a minimum, tyrosine kinase inhibitors such as genistein impaired the histamine H_1_ receptor-mediated positive inotropic effect of histamine [80].

Typically, one assumes that the histamine H_2_ receptor directly increases cAMP by stimulating adenylyl cyclase 5/6 via stimulatory G proteins [2,110]. However, several publications now argue that histamine H_1_ receptors can lead to elevated cAMP in cells and isolated cardiac preparations. For instance, there is a translocation of beta gamma subunits of a stimulatory Gs protein and the alpha subunit of a stimulatory Gs protein that independently induce a cAMP increase, at least in cell lines stably transfected with the human histamine H_1_ receptor [111].

1,2-Diacylglycerol (DAG) activates PKC (Figure 1). This kinase phosphorylates and activates various cell-type-specific target proteins. In non-cardiac cells, histamine H_1_ receptor stimulation via PKC activates ion channels such as potassium or calcium channels (discussed in [43]).

Likewise, inhibitory G proteins couple histamine H_1_ receptors to ion channels in equine tracheal myocytes. This coupling could be interrupted by applying a selective inhibitory antibody against this G protein [19]. This is evidence, even in native cells, that histamine H_1_ receptors can also mediate signalling through inhibitory G_i_ proteins.

In adrenal cells, histamine (10 µM) stimulated the Ca^2+^ transients and increased phosphoinositide formation. This phosphoinositide formation was barely affected by 10 µM mepyramine but nearly abolished by 10 µM clemastine (Table 2). This would argue for the cell type-dependent affinity of histamine H_1_ receptor antagonists [112]. Significantly, in a clonal CHO cell line, the guinea pig histamine H_1_ receptor was stably expressed and used to assess signal transduction [50]. In these CHO lines, all second messenger systems described previously in various tissues could be recapitulated. H_1_-histamine-receptors could increase in CHO cells Ca^2+^ transients, activate PLC leading to phosphoinositide production, could activate PKC and increase the cellular content of AA [50]. The increases in Ca^2+^ transients were independent of extracellular Ca^2+^ and pertussis toxin insensitive [50]. The activation of PLC required extracellular Ca^2+^ and was also pertussis toxin insensitive. The increase in AA was mediated by phospholipase A2 and also required extracellular Ca^2+^ but was in part pertussis toxin sensitive [50].

Histamine H_1_ receptors increase cGMP levels in neuronal cells [113,114]. In this neuronal cell line, histamine H_1_ receptor stimulation did not raise Ca^2+^ levels (measured with aequorin) but increased cGMP levels nonetheless [47]. These cGMP increases were attenuated by 100 μM of quinacrin [47]. Their interpretation was that the histamine H_1_ receptor activated phospholipase A (inhibiting by quinacrin), which then raised levels of AA, leading to increased cGMP levels [47]. AA then probably entered the lipoxygenase pathway because nordihydroguaiaretic acid, an inhibitor of lipoxygenase activity, abolished any formation of cGMP [47]. Cyclooxygenases were apparently not involved, as indomethacin did not inhibit cGMP formation (Table 4, [47]). In addition, NO, in the cells where the NO was produced (smooth muscle cells, endothelial cells, or cardiomyocytes) or in surrounding cells such as cardiomyocytes, can activate NO-dependent cytosolic GC that produce cGMP (Figure 1). cGMP can then activate phosphodiesterase 2 (PDE2), which reduces cAMP levels. Higher concentrations of cGMP in human atrial cardiomyocytes can, in contrast to lower concentrations of cGMP, decrease PDE3 activity and raise cAMP levels [115] and thereby can increase cAMP-mediated effects, leading to an elevation in force of contraction and might augment the beating rate of isolated right atrial preparations or the beating rate of perfused whole hearts.

Histamine H_1_ receptors can lead to the phosphorylation of glycogen synthase kinase 3 in some non-cardiac cell lines [116]. In some cell lines, histamine H_1_ receptors can activate Rho and Rac small GTPases via Gq and PLC [38]. Histamine H_1_ receptors induced a time-dependent reversible phosphorylation of ERK that peaked after 5 min with 100 µM histamine [117]. At 3 h of stimulation, 100 μM histamine increased the mRNA of the inflammatory proteins interleukin 8 (IL8) and cyclooxygenase 2 (COX2) [117].

Stimulation of histamine H_1_ receptors in transfected HeLa cells for 24 h led to increased activity of some transcription factors (SRE, MYC response element, Figure 1) and the protein expression of MYC but decreased the phosphorylation state of β-catenin (within 5–14 min, [116]). In HeLa cells, histamine (at 1 µM and higher) also increased the phosphorylation state of GSK-β3 (within 5 min [116], Figure 1). Histamine H_1_ receptors could induce within minutes the protein expression of Rho, Ras, ERK1/2 and Jun in histamine H_1_ receptor-transfected CHO cells [118]. These effects were blocked by 10 µM U73112, a PLC inhibitor [118]. Longer-lasting stimulation of histamine H_1_ receptors can increase the transcription of NOS in endothelial cells [119]. This is, in last consequence, the result of increased Ca^2+^ the cytosol of the cell, but perhaps also in the nucleus of the cell. In any case, this histamine H_1_ receptor-mediated augmentation of the Ca^2+^ concentration within the target cell increased the activity of a Ca^2+^-dependent protein kinase called CamKinase II [119]. It has been suggested that in hypoxia, the enhanced activity of NOS would lead to the generation of detrimental free radicals from mitochondria in the heart [119]. In addition, for instance, in the brain and adrenal glands [120], stimulation of histamine H_1_ receptors can increase cAMP levels indirectly via increased Ca^2+^ and stimulation of adenylyl cyclase 1 or 3, or via Gs stimulation of adenylyl cyclase 5 and 6.

For the human heart, some controversy exists about the expression of adenylyl cyclases. For example, some detected nine adenylyl cyclases, except for adenylyl cyclase 8, in the human heart [121]. Later, adenylyl cyclase 3 was reportedly lacking in the human heart [110]. In a recent study, adenylyl cyclase 1, 2, 3, 4, 5, 6, 7, and 8 were detected using polymerase chain reaction (PCR) but also immunologically in cardiomyocytes, at least in rat hearts [120]. Ca^2+^ can now stimulate adenylyl cyclase 1 and adenylyl cyclase 3 [110,122], expressed in the human heart. Thus, histamine H_1_ receptors could stimulate Ca^2+^ levels in cardiomyocytes and consequently would be able to stimulate adenylyl cyclase 1 or adenylyl cyclase 3 to generate more cAMP in cardiomyocytes and increase the force or beating rate (Figure 1). Interestingly, in principle, the receptor composition in the bovine adrenal gland is similar to that in the human atrium [123]. In bovine adrenal cells, both histamine H_1_ and H_2_ receptors are functional. In brief, 5 µM histamine can increase cAMP levels. This increase can be partially antagonised by 1 µM mepyramine, in part by 1 µM cimetidine, and entirely by combining mepyramine and cimetidine [124]. The histamine H_1_ receptor agonist 2-thiazolylethylamine (Table 1) likewise increased cAMP levels in adrenal cells. The activation effect of histamine H_1_ receptors depends on sufficient high extracellular Ca^2+^ levels for these cells [124]. The effect of histamine H_1_ and H_2_ receptor stimulation on cAMP levels in adrenal cells was more than additive [124]. This suggests a synergism of histamine H_1_ and H_2_ receptors that might also exist in the human atrium. Histamine H_1_ receptor stimulation does not raise cAMP levels by itself [50]. In contrast, histamine H_1_ receptor stimulation further increased a forskolin-induced cAMP increase in the CHO cells [50]. This effect was not dependent on extracellular Ca^2+^ and was pertussis toxin insensitive.

In an endothelial cell line and HUVECs, 100 µM histamine increased cytosolic Ca^2+^ levels [76]. This effect was mimicked by 100 µM of a histamine H_1_ receptor agonist called 2-[(3-trifluoromethyl)phenyl]histamine dimaleate (TMPH, Table 1) and was antagonised by mepyramine [76]. This increase in Ca^2+^ was blocked by 500 nM bafilomycin A1 or 1 µM tharpsigargin [76]. In these cells, the Ca^2+^ was mobilised at least in part, by NAADP [76]) (Figure 1).

## 4. Desensitisation, Downregulation and Upregulation of the Histamine H_1_ Receptor

In principle, the histamine H_1_ receptor shows desensitisation, even in isolated cell culture studies. Interestingly, in a study on HeLa cells (a human uterine carcinoma line), 100 µM histamine induced a rapid increase in Ca^2+^ transients that fell steeply, followed by a second sustained increase in Ca^2+^ transients [58]. We want to point out that these data are consistent with a biphasic functional response to histamine H_1_ receptor stimulation. After 5 min of stimulation with 100 µM histamine and washout, a second stimulation with 100 µM histamine revealed that the initial steep and the subsequent shallow increase in Ca^2+^ transients were reduced by approximately 50%. Desensitisation was reduced when PKC was previously activated (with 0.2 µM phorbol-12-myristate-13-acetate). The desensitisation was more pronounced on the second peak of the Ca^2+^ transient than on the first peak of the Ca^2+^ transient [58]. The desensitisation via PKC was explained by the phosphorylation of amino acid 398 in the histamine H_1_ receptor [125]. In addition, part of the desensitisation of the histamine H_1_ receptor is due to the activation of the G protein-dependent receptor kinase (GRK2 [57]). This was measured as the partial reduction of 100 μM histamine-induced increases in phosphoinositide levels in cells transfected with active and inactive constructs for GRK2 or small interfering RNA (siRNA) for GRK2 [57]. Studying this kind of desensitisation of the histamine H_1_ receptor in the heart would be interesting. Likewise, a histamine H_1_ receptor-dependent potassium current is quickly desensitised in a gastric epithelial cell line [126].

As mentioned above, in the guinea pig paced left atrial preparation, only the histamine H_1_ receptor mediates the positive inotropic effect of histamine (Table 4). No heterologous desensitisation was observed in this tissue. Left atrial preparations were desensitised using high concentrations of beta-adrenoceptor agonist isoprenaline. However, this did not change the potency of histamine to increase the force of contraction in left atrial preparations from guinea pigs [127]. The opposite experiment, trying to desensitise the effect of isoprenaline by desensitisation with a single high concentration of histamine in guinea pig left atrial preparations, is lacking. Moreover, homologous desensitisation of the effect of histamine in guinea pig left atrial preparations has not yet been systematically studied.

Downregulation and internalisation have different molecular mechanisms. Mutating five different serines or threonines to alanines in the second and third intracellular loops and the C-terminus of the human histamine H_1_ receptor in cultured cell lines led to agonist-induced downregulation, not to histamine H_1_ receptor internalisation [128]. One could study such phenomena in transgenic mice, where such mutated receptors are overexpressed in the heart. One can speculate that downregulation is a protective mechanism, and if patients express a mutant histamine H_1_ receptor that is not correctly downregulated, detrimental effects on organ function might be anticipated.

Internalisation is a rapid process, at least in CHO cells transfected with the human histamine H_1_ receptor: 30 min exposure to 100 µM histamine [129]. A relevant amount of the histamine H_1_ receptors was translocated from the cell surface into the interior of the CHO cells and involved GRKs [129]. This internalisation involved clathrin-coated vesicles but did not involve caveolae [129]. Internalised histamine H_1_ receptors were then ubiquitinated and degraded in lysosomes and proteasomes [129].

The reversal of desensitisation (resensitisation) was not due to the reintegration of preformed histamine H_1_ receptors but to the new synthesis of histamine H_1_ receptors [117]. Interestingly, and of potential clinical relevance, there is crosstalk with respect to desensitisation between histamine H_1_ and H_2_ receptors. Histamine H_1_ receptor stimulation in U937 cells reduced the extent to which amthamine (an agonist at histamine H_2_ receptors but not at histamine H_1_ receptors) could increase cAMP levels within 10 min, arguing for crosstalk between histamine H_1_ and H_2_ receptors [39]. GKR2 also causes homologous desensitisation of histamine H_1_ receptors in some cell lines [57,130]. The crosstalk was shown to be GRK2-mediated [39]. It was speculated that internalised dimeric histamine H_1_/H_2_ receptors might activate the MAP kinase cascade via arrestins [39]. It is unclear whether the homologous desensitisation of the histamine H_1_ receptor depends on clathrin or raft structures [129,131]. In crossover experiments, by treating U937 cells with 10 µM amthamine, the extent to which stimulation of histamine H_1_ receptors could increase the Ca^2+^ or IP_3_ levels was attenuated [39]. Pretreating U937 cells with 10 µM cetirizine (Table 2) for 30 min attenuated the amthamine-induced increase in cAMP in U937 cells [48].

Likewise, pretreating U937 cells with 10 µM cetirizine for 30 min led to the internalisation of not only histamine H_1_ but also H_2_ receptors in U397 cells [48]. For instance, the inverse agonist mepyramine reduced the lipopolysaccharide (LPS)-induced increase in the mRNA for IL8; this anti-inflammatory effect was attenuated by stimulating histamine H_2_ receptors in these cells (amthamine, 10 µM, [48]). Moreover, treatment with cimetidine (90 min, 10 µM) led to the internalisation of histamine H_1_ receptors in U937 cells. Clinically, that could mean that cimetidine might be useful for treating, e.g., hay fever. A dynamin inhibitor could impair internalisation (dynasore, 80 µM, 30 min). After histamine H_1_ and H_2_ receptor stimulation, heterodimers of histamine H_1_ and H_2_ receptors appeared in the endoplasmatic reticulum of U937 cells [39]. It would be interesting to know whether such heterodimerisation also occurs in the heart, namely the human heart, and can activate MAP kinases.

In humans, there is evidence that upregulation of the histamine H_1_ receptor in disease, in principle, occurs. There was an upregulation in the brain of patients with epileptic seizures and in patients with allergic rhinitis in the nasal mucosa [128,132]. Typically, the stimulation of a receptor by its agonist leads to downregulation. In contrast, under certain experimental conditions, the histamine H_1_ receptor mRNA undergoes upregulation. For instance, when HeLa cells were treated for 3 h with 10 µM histamine, the histamine H_1_ receptor mRNA expression increased. This upregulation involved PKC delta because inhibiting this kinase blocked the upregulation of the histamine H_1_ receptor [133]. This upregulation is clinically detrimental. Hence, the finding that inverse histamine H_1_ receptor antagonists alone or in the presence of histamine, at least in HeLa cells, reduce the mRNA of the histamine H_1_ receptor is relevant [59]. Upregulation in HeLa cells was transcriptionally regulated as the stability of the mRNA was not increased [134]. Upregulation involved the activation of PKC, which occurred first on mRNA (around one hour after histamine treatments) and then on protein levels (after four hours [134]). This upregulation was inhibited by antagonists of the histamine H_1_ receptor, which was regarded as evidence that this might be the mechanism for the therapeutic benefits of histamine H_1_ receptor antagonists in chronic allergic diseases [134].

Upregulation of histamine H_1_ receptors in smooth airway muscle cells has also been reported. Two hours of stimulation with the β_2_-adrenoceptor agonist fenoterol and other cAMP-increasing agents upregulated the expression of histamine H_1_ receptors on mRNA and protein levels [135]. The mechanism consisted of an increased transcription rate and enhanced mRNA stability. More relevant was that histamine was now more potent in inducing smooth muscle contraction. The authors suggested that their findings might explain the declining clinical effect of prolonged inhalation of β-adrenoceptor agonists in asthma patients [135]. In heart failure, the level of cAMP in the heart declines. Hence, one might predict by extrapolation that the density and function of histamine H_1_ receptors in the heart might decline [136]. However, this is currently speculation. Histamine H_1_ receptors can desensitise. This may be cell- or cell-line dependent. In an alveolar cell line, this is partly due to receptor internalisation [118] and does not involve GRK2, dynamin or β-arrestin [117]. However, at least in other cell lines, GRK 5 and 6 and arrestins seem to be involved [33,57]. Hence, it will be interesting to study whether GRKs are involved in desensitising human cardiac histamine H_1_ receptors.

## 5. Histamine H_1_ Receptor and Endothelial Cells

The histamine H_1_ receptor mediated the vasodilatory effect of histamine in guinea pig coronary arteries [97]. This action is probably due to histamine H_1_ receptors on vascular endothelial cells. In vascular endothelial cells, stimulation of histamine H_1_ receptors leads to the contraction and shortening of endothelial cells to increase the permeability of the vessel walls [12]. The signals that the histamine H_1_ receptor conveys into the vascular endothelial cells have already been mentioned in general terms (Figure 1). The signals comprise the stimulation of phosphoinositide formation and the formation of prostacyclins. As a function of the endothelial cells after histamine H_1_ receptor stimulation, platelet-activating factor and von Willebrandt factor are secreted [12]. As mentioned above, for other cell types, histamine H_1_ receptors can increase the formation and, thereafter, the release of nitric oxide in vascular endothelial cells [12] (Figure 1). Stimulation of IP_3_ receptors in endothelial cells increased the release of Ca^2+^ from intracellular stores (Figure 1).

This elevated Ca^2+^ can activate several Ca^2+^-stimulated enzymes in vascular endothelial cells. In cultured bovine thoracic endothelial cells, 200 µM histamine-induced NO production in a Ca^2+^-dependent fashion [137]. More clinically relevant, histamine elevated intracellular Ca^2+^ and activated NO production in cultured human umbilical vein cells (HUVEC, [136]). However, the authors showed that the histamine H_1_ receptor desensitised but not increased NO production and raised the possibility that Ca^2+^ may only partially enable increased NO production [136]. Specifically, extracellular Ca^2+^ and a polarised cell membrane are necessary for stimulating NO production [136]. The authors speculated that the histamine H_1_ receptor might open Ca^2+^ influx through the membrane as a trigger for NO production. The histamine-dependent increase in Ca^2+^ was attenuated by cAMP-increasing agents such as forskolin. Histamine led in human umbilical vein endothelial cells (HUVEC) to an increase in cGMP, as expected (Figure 1) [138].

## 6. Histamine H_1_ Receptor and Smooth Muscle Cells

Stimulation of histamine H_1_ receptors on smooth muscle cells can lead to vasoconstriction. The histamine H_1_ receptors on smooth muscle cells in the coronaries elevate cytosolic Ca^2+^ levels that lead to contractions. In coronary endothelial cells, histamine H_1_ receptors likewise elevate Ca^2+^ levels. This increase has opposite functional consequences; histamine H_1_ receptor stimulation leads to nitric oxide (NO) formation. This NO diffuses out of the endothelial cells to neighbouring smooth muscle cells, causing vasodilatation. Histamine H_1_ receptors are present and functional in smooth muscle cells. This is relevant, especially in the vessel walls of coronary arteries. Stimulation of these histamine H_1_ receptors leads to vasoconstriction. The underlying mechanism is not entirely understood. However, it seems clear that the initial step is the coupling of the histamine H_1_ receptor via a G protein (probably G_q_) to phospholipases. Their activation generates DAG and IP_3_. The IP_3_ releases Ca^2+^ from the smooth muscle endoplasmatic reticulum.

Thereafter, Ca^2+^ in the cytosol of smooth muscle cells increases. In part, this Ca^2+^ can directly interact with myofilaments to induce vasoconstriction. Then, Ca^2+^ activates Ca^2+^-dependent protein kinase and protein phosphatase (calcineurin) cascades. For instance, Ca^2+^ can activate myosin light chain kinases, which lead to the phosphorylation of myofilaments and thus contribute to contraction. The role of histamine receptors on Ca^2+^ in the smooth muscle has been reviewed elsewhere for the interested reader [2,3]. Furthermore, DAG could activate the various isoforms of protein kinase C (PKC) and lead to the translocation of the isoforms of PKC to various compartments relevant to vasoconstriction. In all likelihood, additional pathways independent of G proteins, such as arrestins, also contribute to vasoconstriction. It seems clear that besides vasoconstriction, histamine H_1_ receptors in the smooth muscle regulate many other understudied functional processes, such as mitochondrial function and gene transcription. Receptor binding studies have revealed histamine H_1_ receptors in bovine aortic smooth muscle cells [139]. The vasoconstrictory effects of histamine H_1_ receptor stimulation in rabbit coronary arteries were accompanied by a decrease in cAMP levels measured in these arteries [140]. This was interpreted as evidence of a direct or indirect inhibitory action of histamine H_1_ receptors on adenylyl cyclase activity [140].

## 7. Histamine H_1_ Receptor in Cardiac Fibroblasts, Cardiac Mast Cells and Cardiac Blood Cells

Mast cells are present and sessile in the human heart. Mast cells are the primary source of histamine in body cells. It has been reported that antagonists at histamine H_1_ receptors reduce the release of histamine and other mediators from mast cells [7]. It is clear that these effects on mast cells are partly independent of the inhibition of histamine H_1_ receptors, but other chemical properties of H_1_ antihistaminergic drugs play a role [7]. Moreover, in cell systems, antihistaminergic drugs can inhibit free radical generation in vitro. However, these effects are probably not histamine H_1_ receptor-mediated but due to ancillary effects of the molecules because mast cells do not express histamine H_1_ receptors [7]. The density of mast cells increases in heart failure and cardiac stress [141,142]. More specifically, the density of mast cells in the human heart is elevated in patients with ischaemic and idiopathic cardiomyopathy [143]. Mast cells in the human heart often reside near nerve endings [111]. The release of mediators, including histamine, from human cardiac mast cells has been suggested to cause arrhythmias [143]. In ischaemia and reperfusion, free radicals are formed, which can release histamine from cardiac mast cells [143]. Mediators released from mast cells can induce cardiac fibrosis.

Hypertension in spontaneously hypertensive rats was accompanied by increased cardiac mast cells. Giving nedocromil, a mast cell stabiliser, is expected to inhibit the release of histamine from mast cells and reduce cardiac fibrosis. In animal models, mast cells were recruited from fat tissue into the heart in myocardial infarction. As with mast cells, basophils contain histamine and are recruited in cardiac inflammation into the heart. Histamine H_1_ receptor antagonists can reduce the production of these proteins in basophils and mast cells [144]. Mast cells occur in the human heart and are increased in heart failure [145]. On the surface of mast cells, histamine H_4_ receptors were identified, which are supposed to inhibit cAMP levels and increase histamine release from mast cells. In contrast, histamine H_2_ receptors increase cAMP levels and inhibit histamine release from mast cells. The affinity of histamine for histamine H_2_ receptors is lower than for histamine H_4_ receptors [1]. Thus, one has speculated that once low concentrations of histamine activate histamine H_4_ receptors, histamine is released from mast cells. Released histamine now has a higher local concentration near the plasma membrane of the mast cells, where it now activates low-affinity histamine H_2_ receptors and, in a negative feedback loop, inhibits the further release of histamine from mast cells [111]. Histamine released from mast cells can stimulate histamine H_1_ receptors on nearby T cells and alter their function [123].

Histamine H_1_ receptors are present in T-helper cells [146]. At least in leukocytes, histamine H_1_ receptor stimulation can decrease the cAMP level by increasing PDE activity [147]. This increase could be due to kinase-induced phosphorylation and the activation of a PDE [147].

At least in skin fibroblasts, histamine H_1_ receptors were detected as protein and mRNA [148]. In skin fibroblasts, histamine H_1_ receptors stimulate phosphoinositides, leading to a transient increase in intracellular Ca^2+^ and altered activity of transcription factors [148,149]. Histamine H_1_ receptors in fibroblasts generally increase collagen production and can lead to fibrosis. One might speculate that targeting histamine H_1_ receptors should be helpful to prevent or treat fibrosis that contributes to, e.g., diastolic heart failure.

A histamine H_1_ receptor-mediated anti-inflammatory signal encompassed NFκB. Histamine can activate the transcription factor NFκB in many cell types. NFκB is well studied. It translocates from the cytosol to the nucleus, binds there to the DNA, and activates the transcription of pro-inflammatory proteins (IL1β, IL6, TNFα; iNOS, VAM-1, iNOS) [7]. In a transfected animal cell model, the activation of an NFκB luciferase gene could be attenuated by all tested H_1_ antihistaminergic drugs acting as inverse agonists [144].

Coxsackie viruses are a relevant clinical cause of myocarditis. In response to this infection and inflammation, histamine H_1_ receptors are beneficial. This conclusion is based on the observation that virus-induced myocarditis is more severe in mice with the histamine H_1_ receptor ablation than in control wild-type mice. The published mechanism is an impaired anti-inflammatory effect of beneficial T cells and an increase in detrimental regulatory T cells [150].

## 8. Histamine H_1_ Receptor and Cardiomyocytes

The expression of histamine H_1_ receptors in wild type mouse (WT) cardiomyocytes was detected using immunofluorescence and specific antibodies [151]. In isolated electrically paced cardiomyocytes from the left atrium of rabbits containing histamine H_1_ receptors [152], one noted an increase in Ca^2+^ transients in the presence of an increasing concentration of histamine (10 nM–10 µM). This increase was blunted by the histamine H_1_ receptor-antagonist chlorpheniramine (1 µM). It was weaker than the increase in Ca^2+^ with an equally effective concentration of isoprenaline with regard to a positive inotropic effect [152]. The authors argued that this rise in Ca^2+^ transients did not originate from the SR because it was not reduced by 10 µM cyclopiazonic acid.

However, the increase in Ca^2+^ transients was, however, blocked by 1 µM nifedipine [152]. These results were interpreted as evidence that stimulation of histamine H_1_ receptors can increase the sensitivity of myofilaments for Ca^2+^ [152]. With the help of antibodies against histamine H_1_ receptors, one has identified histamine H_1_ receptors histologically in cardiomyocytes in the atrium and ventricle of guinea pigs [153]. A higher density of histamine H_1_ receptors can be found in sinus node cells and atrioventricular node cells than in the cardiomyocytes of the surrounding myocardium [153]. This higher expression of the histamine H_1_ receptor in specialised cardiomyocytes could lead to a higher affinity for endogenous histamine (or synthetic agonists at histamine H_1_ receptors). Thus, this high cellular density of histamine H_1_ receptors might explain why histamine H_1_ receptor agonists inhibit atrioventricular conduction in isolated human cardiac preparations [101] and in the isolated guinea pig heart [97,154,155].

## 9. Histamine H_1_ Receptor and Cardiac Electrophysiology

In the brain and specific cardiac cells, histamine can activate cells by inhibiting repolarising potassium current(s) in the cell surface [12]. Histamine via histamine H_1_ receptors can prolong the monophasic action potential duration (APD) in multicellular muscle preparations and isolate cardiomyocytes from the left atrium of the guinea pig [94,152,156,157]. This seems contradictory, since activation of repolarising currents should shorten the APD. However, the effect on APD is a combined effect of calcium current and several potassium currents. The net effect depends on how important the calcium current is for the APD. This is a long-standing debate; there are significant differences in this phenomenon between rat cardiomyocytes and guinea pig cardiomyocytes. Moreover, there are also differences regarding this aspect between ventricular, atrial, AV-nodal and sinoatrial nodal cells. Thus, histamine (1–100 µmol/L) in rabbit SA nodes shortened the APD [158].

A minor prolongation of the APD was also noted in guinea pigs’ right papillary muscles using pyridyl-ethan-amine (PEA) (Table 1), which is expected to translate into a positive inotropic effect [156]. However, in isolated AV-node preparations from guinea pigs, only a stimulatory effect of histamine H_2_ receptors was noted, but no inhibitory effect of histamine H_1_ receptor stimulation [156] in contrast to inhibitory effects on AV-node signal propagation in perfused guinea pig hearts [154,155]. In early studies, PEA was given to papillary muscles from guinea pigs in the presence of 10 µM cimetidine to block any effect of PEA on histamine H_2_ receptors [94]. However, PEA is also known to release noradrenaline from multicellular cardiac preparations [159]. Therefore, studies in isolated atrial cardiomyocytes are more convincing (atrial cardiomyocytes: [152]).

In contrast to histamine H_2_ receptors, histamine H_1_ receptors did not increase the current through the L-type-Ca^2+^ channel in multicellular muscle preparations, but also in isolated cardiomyocytes from the left atrium of the guinea pig [152,156,157]. Borchard and colleagues demonstrated in guinea pig left atria a prolongation of APD by histamine, which could be entirely blocked by dimetindene but not by cimetidine, indicating a histamine H_1_ receptor-dependent effect. In voltage-clamp experiments on guinea pig papillary muscles, these authors demonstrated an increase in the slow inward current and net outward current using 1 µmol/L histamine [157]. Histamine (via histamine H_1_ receptors) in isolated left atrial guinea pig myocytes increased cytosolic [Ca]i but did not activate of the L-type Ca^2+^ current [152]. These authors explained that the L-type Ca^2+^ channels are longer opened during the normal action potential because of the inhibition of the repolarising current; therefore, the effect on the L-type calcium channel is indirect.

In contrast, stimulation of the histamine H_1_ receptors in isolated cardiomyocytes from the left atrium of the guinea pig could increase the current through potassium channels, which could explain the measured prolonged action potential [152]. In isolated cardiomyocytes from the left atrium of the guinea pig heart, histamine H_1_ receptor stimulation acted via phospholipase C (Figure 1), as it was inhibitable using 30 nM staurosporin or 100 nM calphostin C. This enhanced the activity of a potassium current named *I*_Ks_, while through another mechanism, another potassium current called *I*_Kr_ was inhibited [43] (Figure 1). Inhibition of *I*_Kr_ or *I*_Ks_ would explain the prolongation of the action potentials of left atrial cardiomyocytes by histamine [43]. It should be noted that *I*_Ks_ is absent in rabbit hearts, so the net effect in rabbits may differ from other species. It was speculated [46] but never reported that this coupling existed only in the atrium but not in the ventricle and might depend on the frequency at which the heart cells beat [43]. From this speculation, one could predict that histamine H_1_ receptors in the guinea pig ventricle partially antagonise the effect of histamine H_2_ receptor stimulation.

At 30 °C bath temperature, histamine via histamine H_1_ receptors prolonged the action potential length in a monotonic fashion, leading to hyperpolarisation in guinea pig left atrial preparation [104]. However, in the same preparation, a puzzling biphasic increase in the force of contraction was noted. The prolongation of the monophasic action potential was not due to altered sodium or calcium currents, but was hypothesised to result from the inhibition of potassium outward currents [104]. While ryanodine (30 nM) failed to affect the prolonged APD after histamine H_1_ receptor stimulation in the guinea pig left atrial preparations, ryanodine attenuated the second phase of the positive inotropic effect of histamine [104]. The effect of ryanodine was explained by an inhibition of Ca^2+^ release from the SR (Figure 1) and suggested an involvement of intracellular Ca^2+^ in the positive inotropic effect of histamine H_1_ receptor stimulation [104]. Later work clarified the situation to some extent. In single atrial cardiomyocytes from guinea pig atrial preparations, 1 µM histamine prolonged the APD (as in the multicellular preparations mentioned before). This action was blocked by 1 µM chlorpheniramine, suggesting a histamine H_1_ receptor-mediated effect [152]. Using a patch clamp protocol, 1 µM histamine did not directly affect the current through L-type calcium channels in guinea pig atrial cardiomyocytes, but inhibited potassium outward currents [152].

Potassium currents in guinea pig atrial cells can be separated at a minimum in *I*_Ks_ and *I*_Kr_. It turned out that histamine H_1_ receptors stimulated *I*_Ks_ via phospholipase C [43]. At the same time, histamine H_1_ receptors inhibited *I*_Kr_ [43]. They argued that *I*_Kr_ is more predominant than *I*_Ks_ in these cells, so the effects do not cancel each other. Still, eventually, the inhibition of *I*_Kr_ overrules and leads to a prolongation of the action potential [43]. In addition, one speculated that histamine H_1_ receptors might lead to alkalisation of the cytosol (increasing the Ca^2+^ sensitivity of the myofilaments) by acting on a sodium/proton exchanger [152]. This has not been studied further. As far as the author could determine, patch clamp experiments on the role of histamine H_1_ receptors in freshly isolated human cardiomyocytes from surgical heart tissue have not been reported. It would be crucial for a deeper understanding of the clinical relevance of histamine H_1_ receptors to study in comparison pacemaker cells, atrial cells, AV-nodal cells, Purkinje cells, and normal ventricular cells with the patch clamp method (e.g., whole-cell patch clamp). Even more challenging but essential would be to study electrophysiological cardiomyocytes from patients with different cardiac pathologies (e.g., arrhythmia, heart failure).

Satoh [158] observed that in sinoatrial preparations from rabbit hearts, histamine (1–100 µmol/L) increased the beating rate, which could be blocked by cimetidine and, thus, exerting a histamine H_2_ receptor-dependent positive chronotropic effect. Electrophysiologically, he showed that histamine could activate the hyperpolarisation-activated inward current *I*_h,_ which was later defined as the funny current *I*_f_, the pacemaker current. However, a detailed patch clamp investigation of histaminergic effects on *I*_f_ (including amplitude, voltage dependence and the activation time constant) is still missing. Clinically, histamine H_1_ receptor antagonists are burdened with the problem of arrhythmias as side effects. Using guinea pig ventricular cardiomyocytes, several potassium channels were inhibited. For instance, *I*_Kr_ (astemizole, terfenadine >> chlorpheniramine, mepyramine), *I*_Ks_ (terfenadine), but also *I*_K1_ (astemizole) were inhibited. This prolongs the APD and can lead to delayed afterdepolarisation and subsequent ventricular fibrillation [160]. However, one must carefully discriminate the effects of histamine H_1_ or H_2_ receptor antagonists alone from the effects of these antagonists in the presence of histamine, since the antagonists may exert their own effects.

## 10. Histamine H_1_ Receptor in Animal Hearts

Usually, it was reported that a positive inotropic effect of the histamine H_1_ receptor was not accompanied and, hence, not mediated by an increase in cellular concentrations of cAMP. As an example of this lack of cardiac cAMP increase, the histamine H_1_ receptor-induced positive inotropic effects in a guinea pig’s left atria can be discussed [81,90,91,92]. A similar histamine H_1_ receptor-mediated positive inotropic effect in the rabbit papillary muscle was also reported [44]. In principle, histamine H_1_ and histamine H_2_ receptors can be found in cardiomyocytes. However, there are substantial species differences in the absolute and relative expression of histamine H_1_ receptors. Moreover, species have profound functional differences, even if histamine H_1_ receptors are present. On the one hand, histamine exerts a positive inotropic effect in isolated atrial or ventricular preparations from humans via histamine H_2_ receptors [161]. On the other hand, cats, rats and mice do not respond to histamine via histamine receptors with a positive inotropic effect, and thus, they might be without much value as models for the human heart [2,3].

In the guinea pig’s left atrium, histamine and 2-(2-pyridyl)-ethane-amine (PEA) exerted a positive inotropic effect via histamine H_1_ receptors [162]. This effect was antagonised by the alkylating agent phenoxybenzamine (better known as an antagonist at adrenoceptors), and phenoxybenzamine led to reduced potency and efficacy of histamine to increase the force of contraction [162]. This was interpreted as evidence of a receptor reserve [162]. Such data are lacking in human atrial preparations. The desensitisation of histamine via histamine H_1_ receptors on Ca^2+^ transients in isolated aortic smooth muscle cells could be attenuated by 10 µM paroxetine, here acting as an inhibitor of GRK2-mediated phosphorylation of the histamine H_1_ receptor [163].

In isolated perfused guinea pig ventricular preparations, histamine induced a positive inotropic effect via histamine H_2_ receptors, also a negative inotropic effect via histamine H_1_ receptors [88,97]. This is seen only if a histamine H_1_ receptor-specific agonist is employed or if histamine is applied in the presence of a histamine H_2_ receptor antagonist. If only histamine is given in the guinea pig ventricle, a concentration- (up to 300 µM without reaching a plateau) and time-dependent positive inotropic effect becomes visible. Interestingly, in these ventricular preparations, PEA exerted a concentration-dependent prolongation of the time to peak tension and the time of muscle relaxation [89]. This was contrary to the effects of histamine H_2_ receptor stimulation (by 4-methyl-histamine), which shortened the time to peak tension and the time of relaxation in the same ventricular guinea pig muscle strips [89].

In contrast, others reported that PEA in the presence of 10 µM cimetidine exerted a concentration- and time-dependent positive inotropic effect in electrically stimulated isolated right ventricular muscle strips from guinea pigs [89]. When the force of contraction was reduced by the L-type Ca^2+^ channel antagonist D600 (0.25 mg/L), the histamine H_2_ receptor agonist 4-methyl-histamine, but not the histamine H_1_ receptor agonist PEA, was able to restore the force of contraction in guinea pig ventricular muscle strips [89]. Similarly, when the force of contraction was reduced using elevated extracellular potassium ion concentrations, additional 4-methyl-histamine, but not PEA, restored the force of contraction [89]. These combined results were correctly interpreted as evidence that the histamine H_1_ receptor, rather than the histamine H_2_ receptor, does not stimulate L-type calcium channels.

However, there is a problem when using PEA in isolated cardiac preparations containing releasable noradrenaline. The positive chronotropic effect of PEA in isolated spontaneously beating guinea pig right atrial preparations and the positive inotropic effect of PEA in guinea pig right ventricular preparations were attenuated by propranolol or reserpine pretreatment, strongly suggesting that PEA can liberate noradrenaline from cardiac stores [159]. Therefore, PEA is also an indirect sympathomimetic agent [159]. There are regional differences. In isolated electrically stimulated guinea pig left atrial preparations, histamine exerted a positive inotropic effect, accompanied by an increase in the production of phosphoinositides [164]. Functionally, this positive inotropic effect was accompanied by a prolongation in time to peak tension and time of relaxation in isolated electrically stimulated guinea pig left atrial preparations, while in the same preparation, isoprenaline, a β-adrenoceptor agonist, shortened time to peak tension and time of relaxation [164]. This argued against a histamine H_1_ receptor-induced cAMP increase, at least in a guinea pig’s left atrium [164]. Even in non-mammalian hearts, one can find reports (e.g., in soft-shelled turtles) in which histamine H_1_ receptors mediated a positive inotropic effect in the isolated heart [106].

Binding data using tritiated mepyramine as a ligand showed somewhat contradictory results. In one study, mepyramine binding indicative of histamine H_1_ receptor expression was noted in guinea pigs’ right atrial and ventricular preparations. However, no binding was measurable in left atrial preparations from guinea pigs [165]. This is difficult to explain because histamine H_1_ receptor stimulation is well known to increase the force of contraction in the left atrium of the guinea pig (Table 4). This problem was readdressed by others [166], who found histamine H_1_ receptors in left atrial membranes from guinea pigs using tritiated mepyramine [166].

These data were again corroborated using Northern and Western blots [167], as already alluded to above. They [167] found that guinea pigs express more histamine H_1_ receptors in the left atrium than in the ventricle, and the situation is the opposite in rabbits. As with guinea pigs and rabbits, the signal transduction of the histamine H_1_ receptor is region-specific; in the ventricle (papillary muscle), histamine H_1_ receptors induce a positive inotropic effect, which was initially judged as IP_3_ mediated. Histamine H_1_ receptors could be identified using ligand-binding experiments and antagonists in the isolated left atrium. These were accompanied by an increase in IP_3_ concentrations but did not contribute to the positive inotropic effects because the levels of IP_3_ increased much later than the increase in the force of contraction [81]. Moreover, in isolated guinea pig left atria, inhibitors of PLC reduced the increase in IP_3_ but not the positive inotropic effects of histamine, arguing against a link between an increase in force via histamine H_1_ receptors and IP_3_ [81]. Similarly, IP_3_ does not mediate the positive inotropic effect of histamine in rabbit left atria [82].

Based on radioligand binding studies and Western blots, the atrium and ventricle from rat hearts contain only a few histamine H_2_ receptors but many histamine H_1_ receptors [105,165]. Nevertheless, histamine lacks any positive inotropic effect in rats [105]. To be more precise, histamine exerted in the isolated rat’s left atrium had a positive inotropic effect, and in the spontaneously beating right atrium, a positive chronotropic effect [105]. However, neither histamine H_1_ nor H_2_ receptor antagonists could block these effects in rats [105]. Fittingly, the inotropic effects of histamine were lacking in rats pretreated with reserpine or when contraction experiments in rat hearts were performed in the presence of β-adrenoceptor antagonists such as propranolol. The contractile effects of histamine in the rat heart are thus, in all probability, a result of a release of endogenous noradrenaline [105]. However, one can ask why histamine H1 receptors were preserved in the rat heart during evolution. We argue that their purpose still needs to be elucidated. A trivial explanation would be that histamine H_1_ receptors are solely expressed in non-cardiomyocytes in the rat heart. Therefore, histamine H_1_ receptors might only be present in smooth muscle or endothelial cells. However, there is data that histamine H_1_ receptors are immunologically present in rat cardiomyocytes [165].

Similarly, in the mouse heart, one found expression of histamine H_1_ receptor mRNA [168]. Nevertheless, histamine exerted no direct effect in the mouse heart but only an indirect effect, such as an indirect sympathomimetic drug of the amphetamine type. When administered as a bolus, histamine exerted positive inotropic and chronotropic effects in wild mouse hearts by releasing endogenous noradrenaline [169,170] but not in the presence of propranolol [13,171,172,173]. If one overexpresses the human histamine H_1_ receptor in the mouse heart, one finds transient negative inotropic, persistent positive inotropic, and negative chronotropic effects [98]. However, in these hearts, overexpression seems to be very high. This can lead to artefacts. For instance, in the case of overexpressed adenosine A_1_ receptors, a gene dosage effect was noted. Stimulating these adenosine A_1_ receptors in low overexpressing atria led to a negative inotropic effect and, at higher levels of overexpression, to a positive inotropic effect [169]. This was explained by a coupling to Gi at low levels of overexpression and Gq at high levels of overexpression [169]. An option would be to use clustered regularly interspaced short palindromic repeats (CRISPR)/Cas technology to make a knockin of the human histamine H_1_ receptor into the mouse genome. In this way, one can assure that no artificial overexpression in the heart occurs. Thus, one could clarify whether human histamine H_1_ receptors are coupled differently from mouse histamine H_1_ receptors to effectors in the heart. In addition, by using an alpha myosin heavy chain promoter, one could be sure that the expression occurs only in cardiomyocytes and compare this mouse model with a knockin model where all mouse histamine H_1_ receptor genes are changed to human histamine H_1_ receptors. It would be exciting to compare their functions. Moreover, one could perform the knockin with a tagged receptor to understand in which cell type the expression has now occurred.

Based on the information above, it seems clear that the density of histamine H_1_ receptors in the heart does not readily correlate directly with their contractile effects. An extreme example is the rat heart. Histamine H_1_ receptors were detected in the rat’s atrium and ventricles using antibodies and Northern blotting [165]. Nevertheless, histamine has no inotropic effect on the rat heart [105]. The same observation was made in wild-type mouse hearts. Hence, in rats and mice, the histamine H_1_ receptors are either not present in the cardiomyocytes and thus no effect would be expected; more interestingly, the histamine H_1_ receptors are present in the cardiomyocyte but do not couple to the force of contraction. The other extreme is the guinea pig’s left atrium or the rabbit’s right ventricle (the papillary muscle). These tissues contain histamine H_1_ and H_2_ receptors at the same protein expression level. However, in the first approximation, only histamine H_1_ receptors couple to the force. Again, one can speculate on which cell type the histamine H_2_ receptors are located, perhaps on non-muscle cells. Or are they on cardiomyocytes, but remain inotropically quiescent? For the guinea pig, it seems likely that the histamine H_1_ receptors do not couple to the force, as they are histologically present in the right atrial cardiomyocytes [165]. The third example lies in the ventricle of the guinea pig. There, both histamine H_1_ and H_2_ receptors contribute to about the same extent as to the force of contraction. The histamine H_1_ receptor can be unimportant for inotropic effects (rats, wild-type mice). The histamine H_1_ receptor can be as critical as the histamine H_2_ receptor (guinea pig ventricle), or the histamine H_1_ receptor can be solely relevant for inotropic effects (left atrium of the guinea pig).

## 11. Histamine H_1_ Receptor in Human Hearts

### 11.1. Histamine H_1_ Receptor in the Human Atrium

Compared to other species, such as rabbits, a high expression of the histamine H_1_ receptor was found in the human heart [165]. Furthermore, the expression of the histamine H_1_ receptor mRNA and protein was significantly lower in the human right atrium than in the human left ventricle [165]. The expression of the histamine H_1_ receptor in the human heart was much lower than in the human cerebrum [165]. This might imply a lesser role of the histamine H_1_ receptor in the human heart than, for instance, in the guinea pig heart. In the guinea pig, the expression in the atrium (the authors did not reveal which atrium they studied) was similar to that in the guinea pig brain [165]. However, these results do not prove a minor role of the histamine H_1_ receptor in the human atrium compared to the guinea pig atrium because there is no positive correlation between tissues. For instance, compared to the rat cerebrum, the rat atrium levels of histamine H_1_ receptors are much higher than in the human comparison. Nevertheless, the rat had no inotropic response to stimulation of the histamine H_1_ receptor [105].

It has been suggested that the expression of the histamine H_1_ receptor in the human heart is transcriptionally regulated [105]. From protein expression data (Western blots [105]), one would expect that a histamine H_1_ receptor-mediated contractile effect would be more prominent in the human left ventricle than in the human right atrium. Data on histamine H_1_ receptor expression in the human left and right atrium are unavailable. While more histamine H_1_ receptors than histamine H_2_ receptors are expressed in the human heart, the positive inotropic effects of histamine in the human heart are mainly histamine H_2_ receptor-mediated because the positive inotropic effects of histamine in the human isolated left atrium or ventricular strips could be wholly antagonised by cimetidine (a histamine H_2_ receptor antagonist, [161]). Similarly, others detected only a positive inotropic effect on histamine in paced human atrial strips, suggesting activation of histamine H_2_ receptors [174] solely. Likewise, no negative inotropic effects of histamine in the isolated atrium or isolated ventricle strips were reported by Ginsburg et al. [161]. 2-(2-aminoethyl) thiazole (ThEA) exerted at 300 µM a positive inotropic effect in human atrial preparations [161].

This effect was regarded as unspecific or histamine H_2_ receptor-mediated [161]. The positive inotropic effect of histamine was not antagonised by mepyramine [161]. Likewise, histamine-induced isolated spontaneously beating trabeculae from the right atrium (of patients with atrial fibrillation) had a positive chronotropic effect, which was cimetidine sensitive [110]. Opposite to the positive chronotropic effect via histamine H_2_ receptors, there is evidence that histamine H_1_ receptors in the human atrium can exert negative chronotropic effects (after blocking histamine H_2_ receptors using cimetidine [101]). Moreover, the positive chronotropic effect of histamine on spontaneously beating human right atrial strips was shifted to lower histamine concentrations using previously applied mepyramine but also carbachol or adenosine [101]. Their interpretation was that histamine exerted a positive chronotropic effect via histamine H_2_ receptors and a negative chronotropic effect via histamine H_1_ receptors that was more pronounced if the cAMP elevating ability of histamine H_2_ receptors was antagonised by stimulation of muscarinic M_2_ or adenosine A_1_ receptors. Only the stimulation of the histamine H_1_ receptor remains [101]. They also reported a negative inotropic effect of histamine in the absence [101] and presence [100] of cimetidine in isolated human atrial preparations in the organ bath.

These adverse inotropic effects were antagonised using a histamine H_1_ receptor blocker and were therefore considered histamine H_1_ receptor mediated. Specifically, they studied both spontaneously beating and with 1 Hz-paced right atrial preparations of patients [100,101]. They constructed concentration–response curves for ThEA. ThEA at 30 µM reduced the force of contraction by 50% [100]. ThEA at 300 µM increased the force of contraction [100]. Studies were repeated in the presence of cimetidine or mepyramine. These antagonist and agonist studies concluded that ThEA (Table 1) at low concentrations stimulated human histamine H_1_ receptors, and at higher concentrations, ThEA stimulated histamine H_2_ receptors.

Accordingly, they noted a negative inotropic effect at low concentrations of ThEA in human atrial preparations and a positive inotropic effect at high concentrations of ThEA. They speculated that the histamine H_1_ receptor-induced negative inotropic effect might have been caused by elevated cGMP content (which they did not measure). This cGMP directly or indirectly could have reduced the force of contraction and beating rate (Figure 1). It seems to be a reasonable approach to repeat these studies with more potent and selective histamine H_1_ receptor agonists, as other groups have challenged them. One line of evidence is that cGMP can stimulate PDE2, degrading cAMP, and thus less cAMP can act. The Fischmeister group has shown this mechanism for the human atrial LTCC [115]. Similarly, cAMP might be reduced in sinus node cells; less cAMP binds to HCN, and bradycardia ensues. At least in mouse sinus nodes, this pathway exists [170].

Furthermore, Sanders et al. [99] did not measure a negative inotropic effect on histamine in the presence of famotidine (a histamine H_2_ receptor antagonist, Table 2) in isolated electrically paced atrial preparations from patients (that is, they did not study spontaneously beating atrial multicellular preparations). Likewise, Zerkowski et al., 1993 [175] did not report a negative inotropic effect of histamine in the presence of diphenhydramine, an antagonist at histamine H_1_ receptors (Table 2). However, histamine increased both cAMP and cGMP levels and stimulated the activity of the cAMP-dependent protein kinase. The increased cGMP levels after 100 µM histamine were abolished entirely using an additional 1 µM mepyramine. Therefore, the increase in cGMP was histamine H_1_ receptor mediated. The increase in cAMP levels and PKA ratio (a parameter for enzyme activity) was not abolished by 1 µM mepyramine but only attenuated to levels seen with histamine (100 µM) alone.

Thus, the increase in the cAMP and PKA ratio was probably mediated by H_2_ receptors. They also described a positive inotropic effect in isolated right atrial preparations from patients via histamine H_1_ receptors [99]. More precisely, they noted that a positive inotropic effect of cumulative histamine could be measured only in preparations from patients who had been treated with β-adrenoceptor antagonists for several weeks. The potency and effectivity of histamine were antagonised by 1 µM of the histamine H_1_ receptor-antagonist mepyramine (Table 2). In contrast, others reported that 1 µM mepyramine increased histamine’s concentration-dependent positive inotropic effect on isolated human atrial preparations [100,101]. Hence, methodological differences or age, medication, comorbidities, mode of anaesthesia or different surgical approaches might account for these conflicting results (Table 5 for details). Surprisingly, they did not use or did not report the effects of specific agonists on histamine H_1_ receptors used by Genovese et al. (Thea and PEA, Table 1).

From their findings, they concluded that histamine acted in a positive inotropic way on both histamine H_1_ and H_2_ receptors in the human heart [99]. They also speculated that histamine H_1_ receptors only augmented the force of contraction in the human atrium if, concomitantly, histamine H_2_ receptors were stimulated. In other words, only when the cAMP levels increase through the stimulation of histamine H_2_ receptors can the stimulation of histamine H_1_ receptors raise cAMP levels in cardiomyocytes further and only then does a positive inotropic effect of the histamine H_1_ receptor occur. This would be similar to cells transfected with the guinea pig histamine H_1_ receptor [50]. Only in the presence of forskolin (1 µM, a submaximal concentration that induced a slight increase in cAMP in cells) could stimulation of the transfected histamine H_1_ receptor increase cAMP levels any further.

The authors proposed two theories to explain the positive inotropic effect of histamine in human atrial preparations that agreed with their biochemical measurements. For one, the histamine H_1_ receptor might increase cGMP levels; cGMP is known to inhibit the activity of phosphodiesterase 2. This would result in increased cAMP levels, and increasing the force of contraction. Alternatively, they speculated [99] on the interaction of histamine H_1_ and H_2_ receptors in the human atrium. They suggested that the histamine H_1_ receptor might attenuate the histamine H_2_ receptor-dependent positive inotropic effect due to the increase in cGMP. They speculated that cGMP elevation might stimulate phosphodiesterase 3, attenuating the histamine H_2_ receptor-mediated cAMP increase [99]. However, there is still debate about where the histamine H_1_ receptor is located in the human myocardium. While histamine H_1_ receptors were quantified using antibodies in Western blot or as mRNA in the human atrium and ventricle [165], these data were obtained with homogenates from human hearts.

Therefore, it is conceivable that histamine H_1_ receptors are not expressed in cardiomyocytes but in other cell types present in the normal heart, such as endothelial cells, mast cells, fibroblasts and smooth muscle cells. One might raise the hypothesis that H_1_ receptor stimulation releases, for instance, prostaglandins from endothelial cells, which diffuse to neighbouring cardiomyocytes to raise the force of contraction. There is precedence for this mechanism concerning the positive inotropic effect of carbachol via muscarinic M_3_ receptors on endothelial cells. Histamine H_2_ receptors, in contrast to histamine H_1_ receptors, can activate cardiac adenylyl cyclase via G_s_ proteins and PLC activity via G_i/q_. Both processes can explain why histamine H_2_ receptor stimulation can increase free Ca^2+^ levels in the cell [176]. As mentioned above, histamine can elevate cAMP levels via histamine H_2_ receptors in isolated human atrial preparations, activate PKA and increase the phosphorylation state of phospholamban [36,99].

### 11.2. Histamine H_1_ Receptor in the Human Ventricle

In humans, no immunohistology of the expression of histamine H_1_ and H_2_ receptors in any region of the heart has been reported. There are few controversial data on the functional expression of histamine H_1_ receptors in the human ventricle. There is convincing evidence that the histamine H_1_ receptor exists in the human ventricle. The histamine H_1_ receptors were detected by Western blotting in human ventricular homogenates [165]. Western blots of G protein-coupled receptors must always be regarded critically. The specificity of the antibodies is crucial and not easy to prove in native human tissue. One can perform blocking experiments, but they can be misleading. It might be helpful in future research to identify the mRNA for the histamine H_1_ receptor with in situ hybridisation and spatial resolution that allows the identification of single cells of the human left ventricle. Alternatively, one could isolate and purify human ventricular cardiomyocytes and use them for Western blots or mRNA measurements; then, the histamine H_1_ receptor must be derived from human ventricular cardiomyocytes. It is quite certain, but never formally, biochemically or histologically proven, that histamine H_1_ receptors are expressed on human cardiac ventricular smooth muscle cells or human cardiac ventricular endothelial cells. However, functional evidence for their presence comes from human coronary blood flow or contraction studies in isolated human coronary atrial rings [161].

The function of these histamine H_1_ receptors in the ventricle on the force of contraction remains uncertain. In early preliminary contraction studies, a positive inotropic effect of histamine was noted. However, it was antagonised in muscle strips from the right and left human ventricles using cimetidine, and ThEA was ineffective [161]. Much later, Du et al., 1993 [102] found a transient negative inotropic effect to histamine in some human left ventricular muscle strips, but only in half of the patients studied. Some data were also presented [89] that may be interpreted as a negative inotropic effect of histamine H_1_ receptor stimulation in human ventricular preparations. To be more precise, they noted in electrically stimulated human ventricular muscle strips that adenosine itself and an adenosine A_1_ receptor agonist attenuated the positive inotropic effect of histamine. They interpreted this finding as unveiling a negative inotropic effect of the endogenous human myocardial histamine H_1_ receptor because stimulation of adenosine A_1_ receptors would abolish or at least attenuate a simultaneous positive inotropic effect of histamine on cardiac histamine H_2_ receptors. They argued that it would be expected that H_2_ receptors should stimulate the activity of adenylyl cyclases (AC), which would be attenuated by adenosine A_1_ receptor stimulation.

However, they did not check whether this ventricular effect of histamine could be blocked by a histamine H_1_ receptor antagonist (such as mepyramine) or could be induced by a histamine H_1_ receptor agonist (such as ThEA). For the human ventricle, at least one publication reported a direct negative inotropic effect of histamine, probably via histamine H_1_ receptor stimulation. However, this negative inotropic effect under conditions specific for histamine H_1_ receptors stimulation was only seen in approximately half of the patients, but clinical details were not given [102]. Moreover, NIE were noted when they generated a cumulative concentration–response curve. Not at low concentrations of histamine, but at intermediate concentrations, they sometimes noted negative inotropic responses to histamine. In fairness, it seems reasonable to assume that the positive or NIE of histamine H_1_ receptor stimulation in the human heart might be due to hidden variables. In other words, it seems worthwhile to perform further studies in human tissue, but with extreme care for the drug and disease anamnesis of the patient.

Conceivably, the operation technique, or how long it took to take the sample from the theatre to the laboratory, might be relevant. Age and gender also explain the conflicting reports on histamine H_1_ receptors in the human heart. Because of the lack of a model system, it is unclear which steps are used in human cardiomyocytes. For this purpose, human ventricular stem cells might be helpful. Indeed, in human cardiac progenitor cells in culture, the expression of the histamine H_1_ receptor was reported on mRNA and protein levels [177]. Simulation of these histamine H_1_ receptors increased Ca^2+^ oscillations, which were attenuated when one blocked the activity of PLC and IP_3_ receptors [177]. This might mean (Figure 1) that, at least in this human model system, a histamine H_1_ receptor is active. However, it remains enigmatic why an increased Ca^2+^ homeostasis should lead to a negative inotropic effect (Table 4); a positive inotropic effect might be expected. Moreover, these results seem dependent on the human heart cells studied. Another group working with human embryonic stem cells failed to detect an increase in intracellular Ca^2+^ after stimulation with histamine [178]. The best system would be isolated primary human adult atrial and ventricular cardiomyocytes, but only in a few laboratories because considerable expertise is required and tissue must constantly be available.

In vivo, histamine infusion decreased left ventricular contractility in healthy volunteers, partly stimulating histamine H_1_ receptors [174]. This report is at variance with a later study. Others measured histamine’s predominant positive inotropic effect on muscle strips from the human ventricle via histamine H_2_ receptors [102]. Hence, neuronal effects in living persons might have hidden a direct positive inotropic effect. Moreover, in this study, echocardiography was used instead of direct pressure measurement in the ventricle and, thus, might have overlooked some effects. Other methodological differences could also play a role.

## 12. Histamine H_1_ Receptor in Disease

### 12.1. Arrhythmias

The various ways in which histamine H_1_ receptor-mediated vasoconstriction can induce arrhythmias have already been summarised [179]. Upon stimulation by histamine, normal human coronary arteries respond to histamine mainly via histamine H_2_ receptors on smooth muscle cells and histamine H_1_ receptors on endothelial cells with vasodilatation. However, human epicardial vessels in coronary arteries respond even in normal patient samples with vasoconstriction. If the endothelium is harmed due to a disease process, vasoconstriction in human and animal coronary arteries occurs [180,181,182,183]. This vasoconstriction can be reversed by the application of histamine H_1_ receptor antagonists. Vascular smooth muscle contraction was shown to be caused by stimulation of non-selective cation channels and membrane depolarisation, leading to the activation of L-type calcium channels [184].

In the guinea pig’s perfused heart, histamine H_1_ receptors could reduce the threshold for ventricular fibrillation, suggesting an essential role in cardiac arrhythmogenesis [145,154,185]. Histamine H_1_ receptors were, at least in the organ bath, not arrhythmogenic in the human atrium. Arrhythmias in human atrial preparations in the organ bath could not be antagonised by mepyramine [99]. This seems plausible; only cAMP-increasing agents such as β-adrenoceptor agonists but not IP_3_-increasing agents such as alpha-adrenergic agonists lead to cardiac arrhythmias. For example, one could easily treat H_1_-TG mice for a prolonged period with β-adrenoceptor antagonists to test this theory. At least in infarcted canine ventricles, histamine induced arrhythmias via histamine H_1_ receptors [186]. Interestingly, others found that histamine (10–20 µM) can enhance the oscillatory activity of sheep Purkinje fibres, which could be blocked by verapamil [187]. Similarly, histamine (3 µM) increased pacemaker activity in sheep cardiac Purkinje fibres associated with the shortening of the APD [188]. However, this effect, which can be considered proarrhythmic, was H_2_ receptor-driven in both studies [187,188].

### 12.2. Sepsis and Inflammation

Some data have revealed the role of histamine H_1_ receptors in cardiac diseases. The cardiac expression of histamine H_1_ receptors in rabbits was increased after three hours by experimentally induced sepsis (treatment with lipopolysaccharide, [189]). Using the same model, this group reported increased expression of histamine H_1_ receptor mRNA and protein in pulmonary arteries. Using cecal puncture to induce sepsis in mice, antagonists at histamine H_1_ receptors or ablation of histamine H_1_ receptors reduced lung and liver injury [165,167]. In the liver and kidneys of wild-type mice with a cecal puncture and subsequent systemic sepsis, the mRNA for the histamine H_1_ receptor increased. Here, the question may arise on the role of histamine H_1_ receptors in the kidney. Indeed, all histamine receptors are well studied in the kidney [190]. Histamine H_1_ receptors are present on renal vessels but also in the tubuli and the glomeruli of the kidney. In renal disease for instance in nephrotic syndrome, plasma histamine levels are elevated. Thus, histamine might be detrimental for renal function but this remains to be firmly established [190].

These combined data are consistent with the assumption that the histamine H_1_ receptor protects against sepsis. However, it would be conceivable, but it has not been reported that the cardiac histamine H_1_ receptor increases sepsis and possibly reduces cardiac injury. In addition, in a porcine model of sepsis, histamine H_1_ receptors were involved. Symptoms of sepsis were reduced by diphenhydramine, an antagonist of histamine H_1_ receptors [191,192].

This beneficial effect of diphenhydramine was probably mediated by the vascular effect, because direct cardiac effects were not evaluated [191]. The cardiac effects of histamine in pigs have been studied [90]. However, the positive inotropic effects in the isolated porcine atrium and ventricle were histamine H_2_ and not H_1_ receptor mediated, making an interpretation of these findings with antagonists at histamine H_1_ receptors difficult. The effects of immunologically induced myocarditis (using myosin from pigs as an antigen) could be attenuated by an antagonist of histamine H_1_ receptors. This effect was probably due to inhibiting the immune response or mitochondrial effects. It did not come from cardiac protective effects, as wild-type mice do not express histamine H_1_ receptors that are inotropically active [193]. In inflammation, histamine H_1_ receptors are stimulated, causing pro-inflammatory effects, such as an increase in vascular permeability and increased expression of adhesion molecules in endothelial cells [194]. In the atrioventricular node of isolated perfused guinea pig hearts, histamine H_1_ receptors can even abrogate conduction [155]. Sepsis induced by cecal puncture in mice is known to increase blood levels of histamine [167].

In this mouse model, histamine H_1_ receptor mRNA increased in the lung, and pulmonary function declined, consistent with a bronchoconstrictory effect of histamine in the lung [167]. This detrimental lung function was improved by pretreating the mice with oligodeoxynucleotides that inactivated NFκB expression [167]. This might indicate that NFκB leads via gene transcription to functional deterioration in sepsis and might be of therapeutic benefit [167].

### 12.3. Others

An agonism at cardiac histamine H_1_ receptors is thought to play a unique role in cardiac anaphylaxis [195]. In this context, an antigen–antibody reaction (within or outside the heart) releases histamine from preformed stores such as cardiac mast cells, activating cardiac histamine H_1_ receptors [196,197]. This might induce a constriction of coronary arteries via stimulation of histamine H_1_ receptors and direct inhibition of atrioventricular conduction in anaphylaxis [196,197]. Moreover, histamine can lead to bradycardia via histamine H_1_ receptors in sinus node cells. Such bradycardias have also been noted in the context of cardiac anaphylaxis. Interestingly, histamine H_1_ receptors are increased in arteriosclerotic lesions in human vessels. In animal models, the activation of the histamine H_1_ receptor can induce arteriosclerosis. In ischaemia and hypoxia, histamine was released from the heart. High cardiac concentrations of histamine in coronaries have been observed, possibly activating cardiac histamine H_1_ receptors directly, leading to arrhythmias [198]. Furthermore, histamine H_1_ receptors can indirectly induce cardiac arrhythmias. Histamine might activate histamine H_1_ receptors on coronary vessels, thus directly leading to vasoconstriction. This vasoconstriction can lead to decreased cardiac perfusion, a lack of cardiac oxygen, and, thus, further release of cardiac histamine. Indeed, a vicious circle may start.

Such histamine-induced constrictions of coronary arteries have been reported experimentally in rabbit and pig hearts. A clinical correlate of these experimental findings might exist in the so-called Kounis syndrome. The Kounis syndrome is an eponym, based on the name of a Greek cardiologist who described a strong vasoconstriction of coronary arteries in patients which suffered from allergic reactions. Mechanistically, it might be significant that high histamine concentrations in patients suffering from coronary heart disease were noted in the blood. This suggests a connection between histamine, arteriosclerosis, angina pectoris and myocardial infarction. The well-known sequelae of anaphylactic reactions comprise tachycardia, hypotonus and cardiac shock [195]. This connection is supported by the fact that for the treatment of an anaphylactic shock typically accompanied by elevated blood levels of histamine, histamine H_1_ receptor antagonists are used [124]. Besides immunological processes, there are also drugs that can release histamine from mast cells, such as opiates and some narcotic drugs, as well as vancomycin and some X-ray contrast media [199,200]. Other diseases accompanied by high plasma levels of histamine are hypertonus, heart failure, and angina pectoris, but graft versus host reactions after organ transplantations [189,201,202,203,204,205].

The cardiotoxic effect of doxorubicin in WT mice and human-induced pluripotent stem cells (hiPSC) was aggravated by treatment with astemizole. The authors concluded that some of the cardiotoxic effects of doxorubicin were histamine related and could be antagonised by histamine H_1_ receptors on cardiomyocytes. In hiPSC, the stimulation of histamine H_1_ receptors improved their differentiation to myocytes [206]. The underlying mechanism of the histamine H_1_ receptor-mediated effects was similar to those in other cell types and involved the activation of ERK and STAT3 [151]. Histamine-treated hiPSC improved cardiac function after infarction in living mice more than in controls [151].

In allergic rhinitis, the expression of the histamine H_1_ receptor was elevated in mucosal endothelial, epithelial and neuronal cells [23]. This upregulation was reversed by giving the patients histamine H_1_ receptor antagonists. The underlying mechanisms were addressed in HeLa cells and are alluded to here because the same mechanisms might be operative in the heart. One noted that stimulation of HeLa cells with 100 µM histamine via activation of the isoform PKC delta and HSP 90 led to upregulation of the histamine H_1_ receptor mRNA [23,60]. However, in a neuronal cell line, the histamine-induced upregulation of histamine H_1_ receptors involved PLC alpha, suggesting cell-related differences in the signal transduction of the promoter of the histamine H_1_ receptor [23]. Upregulation was transient and started within 60 min [23]. Hence, it would be informative to study the action of histamine in human atrial preparations under the same conditions to see whether this also occurs in the human heart. This is accompanied by, and possibly caused by, phosphorylation of the PKC delta. After that, PKC activates ERK and MEK (but not RAF-1 [23]).

The upregulation of histamine H_1_ receptors in HeLa cells by histamine was attenuated by pretreating the cells with quercetin, a flavonoid widely found in tea and many fruits. The quercetin did not block histamine H_1_ receptor-induced increases in phosphoinositides, but reduced phosphorylation and thus activation of PKC delta and translocation of PKC to the Golgi apparatus [207]. A similar reduction in nasal histamine H_1_ receptors after an appropriate drug stimulus was seen using quercetin in a rat model of pollinosis [207].

## 13. Agonists and Antagonists

From a clinical perspective, treating patients with receptor-specific drugs is therapeutically relevant. Depending on the disease process one wants to affect, having receptor agonists and receptor antagonists at one’s disposal is helpful. Hence, one can finally address the question of drug therapy using the histamine H_1_ receptor as the target of choice.

Antagonists at histamine H_1_ receptors are listed in Table 2. We have also indicated which antagonists are inverse and which are pure antagonists. There is evidence that long-term treatment of patients leads to the loss of efficacy of antagonists at histamine H_1_ receptors [117]. This was explained by desensitisation via internalisation of the histamine H_1_ receptor [117]. Antagonists at histamine H_1_ receptors are mainly prescribed or used against acute allergic reactions caused by histamine such as rhinitis, urticaria and conjunctivitis. In bronchial asthma, sometimes H_1_-histamine receptor antagonists are sometimes used as adjuvants. Likewise, in combination with epinephrine, H_1_-histamine receptor antagonists are used intravenously to treat anaphylactic reactions. Diphenhydramine may be taken to prevent motion sickness and is sometimes used to induce sedation or treat insomnia.

While all the typical antihistamines listed in Table 2 are inverse agonists [35], neutral antagonists have been synthesised and characterised [208,209]. For instance, mepyramine is an inverse agonist. Mepyramine should therefore antagonise any stimulatory effects of histamine on H_1_-histamine receptors. Moreover, mepyramine should inhibit the intrinsic activity of overexpressed H_1_-histamine receptors as an inverse agonist in cell cultures. In mice, cardiac overexpression of the histamine H_1_ receptor would lead to a reduction in histamine H_1_ receptor-induced effects. We are currently testing this hypothesis in depth.

The design of experimental and clinical studies must examine the side effects of these compounds in primary cells or native tissues. Here, it is meant that the effects of histamine H_1_ receptor antagonists act in an agonistic or antagonistic fashion at other G protein-coupled receptors or ion channels. Typical examples are given in Table 2. Most of the compounds listed in Table 2, such as muscarinergic or serotoninergic receptors, are antagonistic. For instance, diphenhydramine potently blocks muscarinic receptors in the whole body and in the isolated heart. Hence, mepyramine potentiates the inhibitory effects of atropine on M_2_-muscarinic receptors and antagonises the effect of cholinergic drugs. This would lead to positive chronotropic effects, not due to mepyramine’s action on histamine H_1_ receptors but action via off-target effects.

Some side effects were so severe that several antagonists were removed from the market because they inhibited repolarising potassium channels. A well-known example is astemizole and its block of hERG channels (review: [7]). This leads to prolongation of the cardiac action potential and, under unfortunate conditions (mutations of channels, altered plasma ion concentrations), to “torsade de pointes”, culminating in deadly ventricular fibrillation.

Interestingly, ongoing or recently finished clinical trials with some histamine H_1_ receptor antagonists exist. Currently, more than 100 clinical trials on mepyramine are listed on clinical trials.gov. None of them explicitly looked for cardiac effects or side effects. For instance, there is a trial where the efficacy of diphenhydramine for the adjuvant treatment of acute migraine is tested (NCT01825941). In another trial, the above-mentioned anti-muscarinic effects of diphenhydramine were the focus (NCT05586477). The investigator tested the hypothesis that patients taking diphenhydramine to treat allergic rhinitis may suffer from hyperthermia, as a blockage to sweat production is expected from the anti-muscarinic effects of diphenhydramine (NCT05586477).

One study (NCT01495858) tested diphenhydramine as an adjuvant to naproxen in post-surgical dental pain. In a study of urticaria (NCT02935699), mepyramine is used as the active control drug (comparator). Mepyramine is surprisingly still tested as a treatment for occasional sleeplessness (NCT02578186). Astonishingly, there are still studies that include chlorpheniramine or dimetindene to treat the common cold and influenza to reduce symptoms (NCT02246166, NCT01448057). Likewise, chlorpheniramine is being studied to reduce the nasal symptoms of COVID-19 (NCT05449405). Interestingly, some cardiovascular indications of histamine H_1_ receptor antagonists are being tested. For instance, clemastine reduces the anaphylactic reaction to protamine and heparin after cardiac extracorporeal circulation (NCT03826004).

Topical cetirizine is being tested against alopecia areata (NCT05803070). The antiallergic effects led to the initiation of studies to treat neuromyelitis optica with cetirizine (NCT02865018).

There is an ongoing study of the clinical benefits of ketotifen in patients receiving percutaneous coronary interventions (PCI) after myocardial infarction. As alluded to above, the hypothesis is that mast cells are detrimental in coronary heart disease, and the inhibition of histamine release from mast cells and the antagonism on the histamine H_1_ receptor to inhibit pro-inflammatory pathways might be of clinical benefit (NCT05511831). Ketotifen is being tested to prevent anthracycline-induced cardiomyopathy. This putative beneficial effect is said to be due to the iron-chelating effects of ketotifen, not mainly due to its H_1_-histamine receptor antagonistic effects (NCT05511831). Others, however, are currently testing ketotifen as an histamine H_1_ receptor antagonist to treat allergic conjunctivitis (NCT00769886).

The use of antagonists in experimental studies and the use of agonists must be carefully designed. Examples are low concentrations of PEA (Table 1) and ThEA (Table 1), which stimulate histamine H_1_ receptors. However, contraction data in guinea pigs and human cardiac preparations have indicated that higher concentrations of PEA and ThEA also stimulate cardiac histamine H_2_ receptors because their positive inotropic effects are blocked by cimetidine. Another example is suprahistaprodifen. It exerts a significant positive inotropic effect in human atrial preparations and in wild-type mice at 1 µM concentrations. However, these effects were abolished by 10 µM propranolol. Hence, suprahistaprodifen is also a noradrenaline-releasing drug, at least in the human and mouse hearts (unpublished observations Neumann, Rayo, Gergs 2022).

In summary, the authors recommend testing the effects in native cardiac tissues using at least two agonists and two antagonists, preferably from different chemical structural classes, to avoid artefacts. The histamine H_1_ receptor is a member of the growing list of receptors that show biased agonism. In other words, dependent on the agonist used, the histamine H_1_ receptor can couple to more than one signal transduction pathway that is, in principle, possible. For instance, (6)-cis-5-phenyl-7-dimethylamino-5,6,7,8-tetrahydro-9Hbenzocycloheptane (cis PAB) and (2)-trans-1-phenyl-3-dimethylamino-1,2,3,4-tetrahydronaphthalene (trans-PAT) CIS PAT and trans-PAP are both agonists at the histamine H_1_ receptor (Table 1). Interestingly, CIS-PAB increased cAMP levels, but trans-PAP increased IP_3_ levels after stimulation of the histamine H_1_ receptor [15]. However, these data were obtained in cell cultures, in CHO cells, and transfected with the guinea pig histamine H_1_ receptor. Whether the same results would be obtained from cardiac tissue, more specifically in human cardiac tissue, remains to be shown. Studying these compounds in human cardiac tissue or isolated human cardiomyocytes would be interesting. New positive inotropic agents might be developed using these compounds as lead structures. The aim would be to find histamine H_1_ receptor agonists that do not raise cAMP levels. This would be the aim because all cAMP-increasing agents lead to potentially detrimental cardiac arrhythmias in human hearts.

The agonists listed in Table 1 are usually only partial agonists, typically much less active in cell culture systems than histamine. Examples are lisuride (marketed as a dopamine 2 receptor agonist), 2-(thiazol-2-yl)ethanamine (2), 2-(3-bromophenyl), 2-pyridylethylamine (PEA), histamine or 2-(3-(trifluoromethyl)phenyl)histamine. Larger molecules were developed as agonists. One would expect larger molecules to be more selective agonists because of the higher demands for molecular recognition. Such drugs are histaprodifen [39] over methylhistaprodifen to suprahistaprodifen. They are thought to be more agonistic than histamine at histamine H_1_ receptors. However, histaprodifens are also partial agonists of histamine H_4_ receptors [210]. This might lead to complications in interpreting the results. Indeed, in cardiomyocytes, the expression of H_4_ receptors has never been reported, so they are probably absent from cardiomyocytes. However, in neuronal structures in the mouse and human heart, H_4_ receptors can be detected. Their function is probably to inhibit the release of noradrenaline from neuronal cells in the heart. Hence, when histaprodifen is applied in native hearts, the results may be due to histamine H_1_ and H_4_ receptor stimulation. Thus, applying a histamine H_4_ receptor antagonist in such studies may be prudent.

## 14. Conclusions

The role of histamine H_1_ receptors in all mammalian cell types, notably in the human heart, is not perfectly understood. Therefore, it is uncertain what physiological or pathophysiological roles these histamine H_1_ receptors play. Moreover, a better understanding of the contractile function of histamine H_1_ receptors in the human heart will enable us to decide whether they might be targets for drug therapy. For instance, would it make sense to block histamine H_1_ receptors to inhibit arrhythmias, or would we provoke arrhythmias? Would we decrease the force of contraction? Will there be differences between the atrium and ventricle? Do agonists at the histamine H_1_ receptor reduce or increase the density of histamine H_1_ receptors in the human heart? What does this mean for treating allergic diseases with antagonists at histamine H_1_ receptors? Will they reduce or increase the cardiac density of histamine H_1_ receptors, and are these changes beneficial, detrimental or simply neutral? From a fundamental perspective, we must better understand the cardiac cellular signalling of the histamine H_1_ receptor in the human heart before we can devise therapies addressing histamine H_1_ receptors.

## Figures and Tables

**Table 3:** Inhibiting the pathways.

Pathway	Inhibitor	Cell Type Studied	References
AC	SQ 22,536	rat cardiomyocytes	Mittra and Bourreau 2006 [45]
Cam kinase II	KN-62	mouse fetal cardiomyocytes	Okazaki et al., 1994 [46]
Cyclooxigenase	indomethacine	murine neuroblastoma cells	Snider et al., 1984 [47]
cGMP	R-cGMP-S	rabbit heart	Hattori et al., 1988 [44]
DG	R59022: increases DAG level	rat cardiomyocytes	Wientzek et al., 1997 [25]
Dynamin	dynasore	transfected U937 cells	Diaz Nebreda et al., 2019 [48]
EPAC	ESI-05	human bronchial smooth muscle cells	Dale et al., 2018 [49]
G_i_	^1^ pertussis toxin, ^2^ guanosine 5′-O-(2-thiodiphosphate)	^1^ CHO cells ^2^ fibroblasts	^1^ Leurs et al., 1994 [50] ^2^ Burch and Axelrod 1987 [51]
Gq	^1^ chromodepsin, ^2^ BIM-46187	^1,2^ various	^1^ Hermes et al., 2021 [52] ^2^ Zhang et al., 2020 [53]
Guanylyl cyclases	H-(1,2,4)-oxadiazolo-(4,3-a)-quinoxalin-1-one (ODQ, 10 μM), methylene blue	rabbit atrium	Hattori et al., 1990 [54]
Lipoxigenase	nordihydroguaiaretic acid	murine neuroblastoma cells	Snider et al., 1984 [47]
Ikr	E-4031	guinea pig left atrial cardiomyocytes	Matsumoto et al., 1999 [43]
Iks	indapamide	guinea pig left atrial cardiomyocytes	Matsumoto et al., 1999 [43]
IP_3_	^1^ 2-aminoethoxy-diphenyl borate (2-APB), xestospongin B 5 µM	^1,2^ rat cardiomyocytes	^1^ Ibarra et al., 2004 [55] ^2^ Sankar et al., 2014 [56]
LTCC	nifedipine	guinea pig atrial cells	Matsumoto et al., 1999 [43]
PKC	^1^ bisindolylmaleimide, ^2^ PMA, ^3^ rottlerin, ^4^ Ro-31-8220	^1^ T-cells ^2,3,4^ HeLa cells	^1^ Iwata et al., 2005 [57] ^2^ Smit et al., 1992 [58] ^3^ Mizuguchi et al., 2012 [59] ^4^ Nariai et al., 2015 [60]
PLC	^1^ U73112, ^2^ staurosporin, ^2^ calphostatin, ^3^ neomycin,^3^ 2-nitro-4-carboxyphenyl-N,N-diphenylcarbamate,	^1^ U937, ^1^ CHO cells ^2^ guinea pig atrial cells ^3^ Rabbit atrium	^1^ Alonso et al., 2013 [39] ^2^ Matsumoto et al., 1999 [43] ^3^ Hattori et al., 1988 [44]
PLA2	quinacrin	murine neuroblastoma cells	Snider et al., 1984 [47]
MAP kinases	SB203580	^1^ leukemic cell line ^2^ mouse cardiomyocytes	^1^ Birkenkamp et al., 2000 [61] ^2^ Moise et al., 2010 [62]
MEK	U0126	HeLa cells	Mizuguchi et al., 2011 [23]
MLC kinase	^1^ ML-7, PIK	^1^ rat heart ^2^ mouse heart	^1^ Bil-Lula et al., 2018 [63] ^2^ Sun et al., 2021 [64]
NAADP	^1^ PPADS ^2^ Ned 19	^1^ rat heart ^2^ mouse heart	^1^ Pustovit et al., 2016 [65] ^2^ Davidson et al., 2015 [66]
NOS	L-NAME	rat heart	Kostić and Jakovljević 1996 [67]
RAF kinase	RAF kinase Inhibitor 1	HeLa cells	Mizuguchi et al., 2011 [23]
RYR	ryanodine	^1^ guinea pig atrium ^2^ mouse atrium	^1^ Hattori et al., 1988 [44] ^2^ Stemmer and Akera 1986 [68]
PARP-1	DPG	HeLa cells	Mizuguchi et al., 2011 [23]
PDE 2	^1^ EHNA, Bay 607550	^1^ mouse heart ^2^ rat cardiomyocytes	^1^ Neumann et al., 2021 [69] ^2^ Castro et al., 2006 [70]
PDE 3	^1^ cilostamide, ^2^ milrinone	^1^ mouse heart ^2^ human ventricle	^1^ Neumann et al., 2021 [69], ^2^ Brown et al., 1986 [71]
PDE 4	1rolipram	^1^ guinea pig heart ^2^ mouse heart	^1^ Ukita et al., 1999 [72] ^2^ Neumann et al., 2021 [69]
PLD	^1^ FIPI 10 nM	^2^ mouse heart	^1^ McDermott et al., 2020 [73] ^2^ Kim et al., 2007 [74]
PKA	^1^ KT5720 ^2^ Rp-8-CPT-cAMPS) ^2^ H89 ^2^ PKI-myr	^1^ U937, ^1^ CHO cells ^2^ human bronchial smooth muscle cells	^1^ Alonso et al., 2013 [39] ^2^ Dale et al., 2018 [49]
PKG	8-bromo cGMP	human aortic smooth muscle cells	Taylor et al., 2017 [75]
Protein kinase G	GF109203 20 μM	U937, CHO cells	Alonso et al., 2013 [39]
SERCA	^1,2^ tharpsigargin, ^1,3^ cyclopiazonic acid	^1^ human endothelial cells ^2^ rat cardiomyocytes ^3^ mouse cardiomyocytes	^1^ Esposito et al., 2011 [76] ^2^ Rogers et al., 1995 [77] ^3^ Kemecsei et al., 2010 [78]
Store operated calcium ion channels	Ni^2+^	guinea pig left atrium	Hattori and Kanno 1985 [79]
Tyrosine kinases	genistein	guinea pig left atrium	Akaishi et al., 2000 [80]

Superscript numbers refer to numbers of the references in the same row.

**Table 4:** Overview of species-dependent positive inotropic (PIE) or negative inotropic effects (NIE) of histamine H_1_ receptor stimulation in regions of the hearts of several species. Note that histamine H_2_ receptor-mediated effects on contractility might also exist [3] but are not listed here for reasons of simplicity. “Langendorff” means that the isolated hearts in this study were retrogradely perfused through the aorta. PEA: Use of the histamine H_1_ receptor agonist PEA (Table 1). H_1_-TG: Mouse with cardiac overexpression of the human histamine H_1_ receptor.

Species	Right Atrium	Left Atrium	Ventricle	References
Dog	^1,2^ PCE, ^1,2^ PIE		^3^ No effect ^4^ AV: negative dromotropic	^1^ Chiba 1977 [84] ^2^ Chiba 1976 [85] ^3^ McNeill 1984 [86] ^4^ Motomura and Hashimoto 1989 [87]
Guinea pig	No inotropic effect, ^3^ NCE, ^9^ NIE	^3,4,5,6,7^ PIE, ^6,7^ PEA	^1^ NIE: ^1^ Langendorff, muscle strips ^2,6,8^ PIE: muscle strips,^2,6^ PEA ^10^ AV: negative dromotropic	^1^ Zavecz and Levi 1978 [88] ^2^ Mantelli et al., 1992 [89] ^3^ Reinhardt et al., 1974 [90] ^4^ Steinburg and Holland 1975 [91] ^5^ Reinhardt et al., 1977 [92] ^6^ Verma and McNeill 1977 [93] ^7^ Amerini et al., 1982 [94] ^8^ Hattori et al., 1994 [83] ^9^ Wilson and Broadley 1981, 1989 [95,96] ^10^ Levi and Kuye 1974 [97]
H_1_-TG	NCE	NIE, then PIE		Rayo-Abella et al., 2022 [98]
Man	^1^ PIE or ^2^ NIE, ^4^ NCE	not done	^3,4^ NIE: muscle strips ^4^ AV: negative dromotropic	^1^ Sanders et al., 1996 [99] ^2^ Guo et al., 1984 [100] ^4^ Genovese et al., 1988 [101] ^3^ Du et al., 1993 [102]
Mouse (wild type)	No effect	No effect	No effect	Gergs et al., 2019 [13]
Pig	No effect	No effect	No effect	Du et al., 1993 [102]
Rabbit	^2,5^ No effect ^7^ PCE	^5^ PIE ^6^ No effect	^1^ NIE: Langendorff ^2,3,4,5^ PIE: muscle strips	^1^ Coruzzi et al., 1979 [103] ^2^ Hattori et al., 1988 [104], ^3^ Hattori et al., 1990 [54], ^4^ Hattori et al., 1994 [83] ^5^ Verma and McNeill 1977 [93] ^6^ Hattori et al., 1991 [82] ^7^ McNeill 1984 [86]
Rat	No effect	No effect	No effect	Laher and McNeill 1980 [105]
Turtle			PIE	Kinawa and Tasaka 1989 [106]

Superscript numbers refer to numbers of the references in the same row.

**Table 5:** Studies in isolated human cardiac tissues.

References	Tissue	Measured Parameters	Agonists and Antagonists Used	Age, Gender	Disease	Medication
Du et al., 1993 [102]	human atrial preparations	isometric force in atrial and ventricular preparations paced at 1 Hz	histamine (1–1000 µM), mepyramine (1 µM), cimetidine (10 µM), propranolol (1 µM), norepinephrine (1–10 µM)	5 males, 2 females, age 11–42 years	healthy organ donors, death from polytrauma	no drugs, only organ preserving buffer
Genovese et al., 1988 [101]	human right atrial appendage, human ventricular papillary muscles	isometric force in spontaneously beating atrial preparations or paced at 1 Hz, papillary muscle strips paced at 1 Hz, Tyrode solution	histamine (1 µM, 100 µM), pyrilamine (1 µM), adenosine 0.1 µM–100 µM), N^6^-cyclo-pentyladenosine (1 nM–10 µM), carbachol (20 nM)	not reported	corrective cardiac surgery, no heart failure	no cardiotonic drug, no anti-arrhythmic, no diuretics
Guo et al., 1984 [100]	human right atrial appendage	isometric force in spontaneous-ly beating atrial preparations or paced at 1 Hz, Tyrode solution	histamine (0.1–100 µM, pyrilamine (1 µM), cimetidine (3 µM), ThEA (0.1–300 µM), impromidine (0.1–100 µM), pindolol (1 nM), norepinephrine (0.1–10 µM)	not reported	bypass surgery, no heart failure	No cardiotonic drugs, no anti-arrhythmics, no diuretics
Sanders et al., 1996 [99]	human right atrial appendage	Isometric force in atrial preparations paced at 1 Hz or 0.5 Hz or 0.2 Hz, Krebs solution with fumarate, pyruvate, glutamate, glucose	histamine (0.1 µM–1 mM), famotidine (0.1 µM, 30 µM), sodium nitroprusside 10 µM, mepyramine (1 µM), CGP 20712A (300 nM),	71 males, 18 females, mean age: 60 years	coronary artery disease, aortic mitral valve disease, mitral valve disease, no terminal heart failure	β-adrenoceptor antagonists (57 patients), no cimetidine or ranitidine, L-type calcium channel blockers, diuretics, nitrates, ACE-inhibitors, antibiotics, allopurinol, aminophylline, amiodarone, aspirin, amitryptiline, corticoids, bezafibrate, carbimazole, diazepam, analgetics, antidiabetics, pravastatin, simvastatin, prazosin, salbutamol, triazolam, warfarin

## Data Availability

Data sharing is not applicable.

## Abbreviations

G_i_:	inhibitory guanosine triphosphate binding protein,
G_s_:	stimulatory guanosine triphosphate binding protein,
G_q_:	G protein binding protein q,
PLC:	phospholipase C,
PLA_2_:	phospholipase A2.
PKC:	protein kinase C,
NOS:	nitrogen monoxide synthase,
*I*_Kr_:	rapidly inactivating potassium current,
*I*_Ks_:	slowly inactivating potassium current,
cGMP:	cyclic 3′,5′-guanosine monophosphate,
DAG:	1,2-diacylglyerol,
PDE:	phosphodiesterase,
RYR:	ryanodine receptor,
MLC kinase:	myosin light chain kinase,
LTCC:	L-type calcium ion channel,
SERCA:	sarcoplasmic reticulum Ca^2+^ adenosine triphosphate (ATP)-ase,
PLD:	phospholipase D,
Cam kinase II:	Ca^2+^-calmodulin-dependent protein kinase II,
AC:	adenylyl cyclase, NAADP: nicotinic acid adenine dinucleotide phosphate,
IP_3_:	inositol 1,2,4, trisphosphate,
PKA:	cAMP-dependent protein kinase,
EPAC:	exchange protein directly activated by cAMP,
MEK:	mitogen-activated protein kinase kinase,
PARP-1:	poly-ADP-ribose polymerase 1,
RAF kinase:	rapidly accelerated fibrosarcoma kinase
L-NAME:	NG-nitroarginine methyl ester,
MAP kinase:	mitogen-activated protein kinase,
CHO cells:	Chinese hamster ovary cells.
